# Endocrine Therapy-Based Strategies for Metastatic Breast Cancer with Different Endocrine Sensitivity Statuses: A Systematic Review and Network Meta-Analysis

**DOI:** 10.3390/cancers14246100

**Published:** 2022-12-11

**Authors:** Jiani Wang, Yiqun Han, Jiayu Wang, Qing Li, Binghe Xu

**Affiliations:** 1 Department of Medical Oncology, National Cancer Center/National Clinical Research Center for Cancer/Cancer Hospital, Chinese Academy of Medical Sciences and Peking Union Medical College, Beijing 100021, China; 2 State Key Laboratory of Molecular Oncology, National Cancer Center/National Clinical Research Center for Cancer/Cancer Hospital, Chinese Academy of Medical Sciences and Peking Union Medical College, Beijing 100021, China

**Keywords:** metastatic breast cancer, endocrine sensitivity statuses, network meta-analysis

## Abstract

**Simple Summary:**

Over the past few years, a great deal of considerable research has been conducted to develop more effective targeted agents for metastatic breast cancer (mBC) patients. This systematic review and meta-analysis of 47 randomized clinical trials identified CDK4/6i + Fulvestrant 500 as the best treatment option in improving efficacy outcomes in endocrine therapy-sensitive (ETS) patients. Chemotherapy had a higher likelihood of therapeutic success in endocrine therapy-resistant (ETR) patients. In ETR settings, with visceral and bone metastases, CDK4/6i +Fulvestrant 500 was the best regimen, while in ETS patients, CDK4/6i + Fulvestrant 500 was the best for bone metastases and CDK4/6i + aromatase inhibitors (AI) for visceral metastases. This study assessed the relative efficacies and provided a rank order of ET-based regimens in the key endpoints PFS and OS for HR+/HER2- mBC patients with different endocrine sensitivity statuses. Different endocrine sensitivity statuses required various optimal treatment strategies, which may provide guidance for clinical practice.

**Abstract:**

Background: Novel endocrine therapies (ETs) and targeted therapeutic regimens have been developed to dramatically improve the outcome of hormone receptor-positive (HR+)/HER2-negative (HER2-) metastatic breast cancer (mBC). Methods: We performed a systematic search with a predefined search strategy in PubMed, Embase and Cochrane CENTRAL databases to perform a network meta-analysis and evaluate the relative efficacies of ET-based treatment regimens in HR+/HER2- mBC patients with different endocrine sensitivity statuses. The study was registered in the PROSPERO database (CRD42021235570). Results: A total of 47 trials (20,267 patients) were included. Analysis of progression-free survival (PFS) in endocrine therapy-sensitive (ETS) patients revealed cyclin-dependent kinases 4/6 inhibitors (CDK4/6i) + fulvestrant 500 mg (Ful 500) (random effect (RE): hazard ratio (HR), 0.46; 95% credibility interval (CrI), 0.27–0.78; surface under the cumulative ranking curve (SUCRA), 0.93; fixed effect (FE): HR, 0.48; 95% CrI, 0.40–0.58; SUCRA, 0.99) to be the best therapy followed by CDK4/6i + aromatase inhibitors (AIs) (RE: HR, 0.53; 95% CrI, 0.40–0.72; SUCRA, 0.86; FE: HR, 0.54; 95% CrI, 0.48–0.61; SUCRA, 0.91). Chemotherapy followed by CDK4/6i + Ful 500 appears to be the most effective option for the endocrine therapy-resistant (ETR) group. Analysis of overall survival revealed CDK4/6i + Ful 500 (SUCRA: 0.99) and AKTi + Ful 500 (SUCRA: 0.87) to be the first-rank regimen for the ETS group and ETR groups, respectively. Conclusion: Our comprehensive analysis suggests that CDK4/6i combined with ETs may be the best treatment option in terms of PFS for ETS patients and chemotherapy for ETR patients with HR+/HER2- mBC. Different endocrine sensitivity statuses required various optimal treatment strategies, which may provide guidance for clinical practice.

## 1. Introduction

Breast cancer is currently the most common malignancy in women worldwide, accounting for approximately 11.7% of all new malignancies [1]. Hormone-receptor-positive (HR+)/HER2-negative (HER2-) breast cancer accounts for approximately 65–70% of mBC. Existing evidence substantiated that the estrogen receptor signaling pathway plays a pivotal role in cancer cell growth and survival in these tumors, and, hence, endocrine-based therapies (ETs) are considered to be one of the most effective systemic treatment cornerstones [2] for these patients, even in visceral metastatic settings. Despite the perceived significant therapeutic effect of ETs, a major therapeutic challenge in treating HR+ breast cancer is to delay or reverse endocrine resistance (ER). A subset of patients (30–40%) eventually acquire resistance to ETs due to prolonged use in the adjuvant setting [3]. 

Over the past few years, a great deal of considerable research has been made to develop more effective targeted agents. In patients with mBC, the main drivers of resistance include ESR1 gene fusion which may require a combination treatment with targeted agents to overcome the resistance and also to minimize the probability of the emergence of resistance in endocrine-sensitive patients [4,5]. Among the other mechanisms of endocrine resistance, targeting high expression of EGFR or HER2 [6,7], phosphoinositide 3-kinase (PI3K)/mammalian target of rapamycin (mTOR) or RAS/RAF/MEK/ERK pathways could also minimize the emergence of ER in patients with mBC [8]. Furthermore, addition of cyclin-dependent kinase 4/6 inhibitors (CDK4/6i) has also been shown to extend the duration of progression-free survival (PFS) and overall survival (OS) in comparison to ET alone in patients with mBC. Given the absence of direct head-to-head comparisons for all available treatment regimens, decision-making guidelines are urgently needed for multiple therapeutic options. 

Previous meta-analyses have taken into account the efficacy of CDK4/6i and ET combinations but were limited in many aspects, such as discovery of appropriate targeted therapies, use of heterogeneity model, non-inclusion of data related to OS, menopausal women and heterogeneity of disease [9,10]. Furthermore, the studies did not perform an extensive efficacy analysis of all possible subgroups of patients with different endocrine sensitivity statuses. Thus, the objective of this network meta-analysis is to perform an extensive analysis and rank the available ET-based strategies (with/without targeted agents) based on their relative efficacies to guide the clinical decision making for patients with HR+/HER2- mBC with different endocrine sensitivity statuses.

## 2. Materials and Methods

### 2.1. Search Strategy and Eligibility Criteria

A systematic search with a predefined search strategy (Supplementary Table S1) consisting of HR status, advanced breast cancer, interested therapeutic regimens and desired endpoints with relevant study design was performed in PubMed, Embase and Cochrane CENTRAL databases. The search was performed from January 2000 till April 2022 and included only studies published in English. We also performed a supplementary search of major conferences in the American Society of Clinical Oncology Annual Meeting, European Society for Medical Oncology Congress and San Antonio Breast Cancer Symposium to retrieve updated survival data from unpublished landmark trials. 

Only phase II/III randomized controlled trials (RCTs) evaluating the efficacy of at least one ET-based regimen for the treatment of patients with advanced or metastatic HR+/HER2- breast cancer were considered for this analysis. This systematic review and network meta-analysis was based on the Cochrane Collaboration Handbook (version 6.1, https://training.cochrane.org/handbook, accessed on 5 October 2022) and registered in the PROSPERO database (CRD42021235570). The PRISMA (Preferred Reporting Items for Systematic reviews) and Meta-Analyses extension for network meta-analysis guidelines were followed to report the present study. The information was recorded by one investigator (H. Yiqun) and is concurrently collected by two investigators (X. Binghe and W. Jiani). Three investigators independently extracted data.

The classification criteria used for defining endocrine sensitivity statuses followed the current international consensus guidelines. Characteristics of endocrine therapy-sensitive (ETS) population include: (1) completion of adjuvant ETs and recurrence after one year of drug discontinuation; (2) no ETs background, including those mBC without any ETs and recurrence after surgery without adjuvant ETs. Characteristics of endocrine therapy-resistant (ETR) population include: (1) Primary ETR: relapse after less than 2 years of adjuvant ETs, or disease progression after less than 6 months of advanced first-line ETs; (2) Secondary ETR: patients with adjuvant ETs for more than 2 years and relapsed within 1 year of discontinuation, or progress on late first-line endocrine therapy for more than 6 months. If not explicitly mentioned in the clinical trial, other information, such as enrollment and subgroup analysis, was used to determine endocrine status.

### 2.2. Study Objectives and Endpoints

The primary objective of the study was to compare the relative efficacies of the available therapeutic options including monotherapy and combination therapy for the treatment of HR+/HER2- mBC. The primary efficacy endpoint was PFS, defined as the time from randomization to progressive disease or death, and the secondary endpoint was OS, defined as the time from randomization until death with any cause. The efficacy analysis was performed based on endocrine status (ETS and ETR). Subgroup analysis based on specific targeted regimens, site of metastasis (visceral and bone-only) and menopausal status was also performed. Multiple reports collection and data extraction were performed by one author and verified by a second author. A third author resolved disagreements. 

### 2.3. Statistical Analysis

Evaluation of quality of the selected RCTs was performed by the investigators using Cochrane Collaboration’s risk-of-bias tool. For each study, the hazard ratio (HR) for PFS and OS along with their 95% credibility intervals (CrI) were extracted from baseline demographics. A Bayesian network meta-analysis was performed using the “Gemtc” 4.2.1 package from R software (version 4.0.4). Within the framework, heterogeneity across studies was tested by I^2^ and the Q statistics along with a forest plot; statistically significant heterogeneity was defined as a *p*-value < 0.1 or an I^2^ statistic > 50%. A random effect (RE) model was performed when I^2^ ≥ 50%; otherwise, a fixed effect (FE) model was applied to quantitate effect size. The surface under the cumulative ranking curve (SUCRA) value was used to rank the effective treatment regimens. Thus, treatments were ranked in a table from best to worst along the leading diagonal [11] with high odds in SUCRA suggesting better ranking [12]. PFS and OS, as defined above for efficacy analysis of treatments based on endocrine status, will be ranked based on the SUCRA value to suggest the most efficacious treatment group. 

### 2.4. Network Geometry

Based on the availability of evidence, networks were constructed for ETS and ETR patients separately. Since the purpose of the network meta-analysis is to provide the relative efficacies of all the available regimens, more than one evidence network was provided for some of the evaluated endpoints and subgroups. In case of two regimens not being accommodated within the major network of evidence, those regimens were excluded from the analyses. For the construction of the evidence networks, comparison was made at the level of drug class. However, additional subgroup analyses at the level of individual drugs were performed for specific drugs as stated above. 

### 2.5. Risk Bias in Individual Studies 

The quality of each RCT included was evaluated by two independent authors using a Cochrane Risk of Bias 2 (RoB 2.0) tool [13,14]. The tool includes (1) randomization process, (2) deviations from intended interventions, (3) missing outcome data, (4) measurement of the outcome and (5) selection of the reported result [13]. Biases are assessed either as “Low” or “High” risk of bias, or can express “Some concerns”.

### 2.6. Publication Bias and Sensitivity Analysis

Egger’s test and Begg’s test were used to assess the potential publication bias. Sensitivity analysis was performed for those which showed publication bias by step-wise removal of single studies (Supplementary Figures S1–S3). 

## 3. Results

### 3.1. Systematic Review and Characteristics

**Figure 1:**
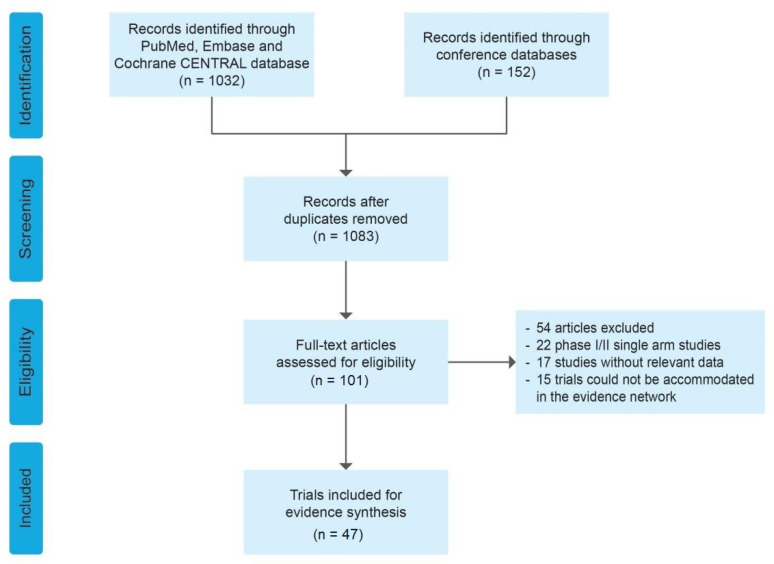
PRISMA flow chart.

A total of 1032 publications and 152 abstract reviews were retrieved through the initial literature and conference search, and 1083 studies remained after duplications were excluded. A total of 47 studies with a cumulative population of 20,267 patients were identified for the detailed analysis. Among the 47 studies, 35 studies were included for the analysis of ETR and 34 studies were included for the analysis of ETS patients (Figure 1). Since the widespread standardized testing of HER2 status was implemented in 2007, articles published from 2009 to 2022 were screened based on the eligibility criteria of this study. The baseline factors of the included studies are provided in Table 1.

### 3.2. Quality of the Evidence

The overall risk of bias of included studies is presented in Supplementary Figure S4. Of the 47 trials included, 33 studies (70%) presented an overall low risk in the five areas of potential bias. A high risk of bias was present in seven (15%) trials while the remaining trials (15%) had “some concerns”, mainly due to open label design and assessment of outcomes by the investigator. The risk of bias was frequently high/unclear in trials with results published only in the form of meeting abstracts (n = 3). 

The methodological quality showed high risk of bias in three trials [29,53,56]. The final results of the ETR OS and ETR of CDK4/6i in combination with other drugs were not affected by the presence of these trials. After removing studies by Jeresaleum et al. [53] and Martin et al. [56], treatment regimens remained the same, confirming that the results were reliable. Similarly, in cases of CDK4/6i in combination with other drugs in ETR and ETS patients, the best treatment regimens remained unchanged after removal of studies [29,56]. There was, however, a noticeable change in the treatment regimens in cases of ETR-PFS and post-menopausal status in ETR patients after exclusion of the trials [53,56].

### 3.3. Analysis of PFS/OS in Patients with ETS

**Figure 2:**
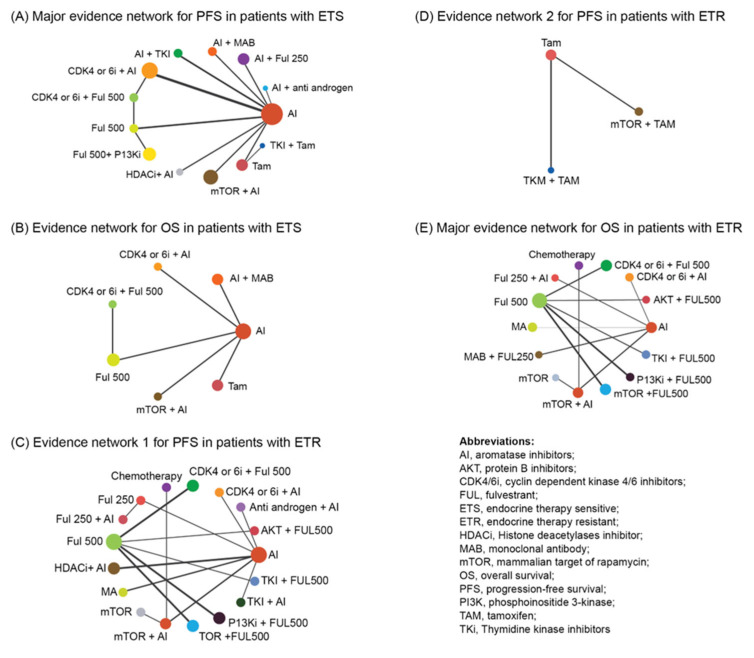
Network meta-analysis plots. (**A**) Major evidence network for PFS in patients with ETS; (**B**) Evidence network for OS in patients with ETS; (**C**) Evidence network 1 for PFS in patients with ETR; (**D**) Evidence network 2 for PFS in patients with ETR; (**E**) Major evidence network for OS in patients with ETR.

**Figure 3:**
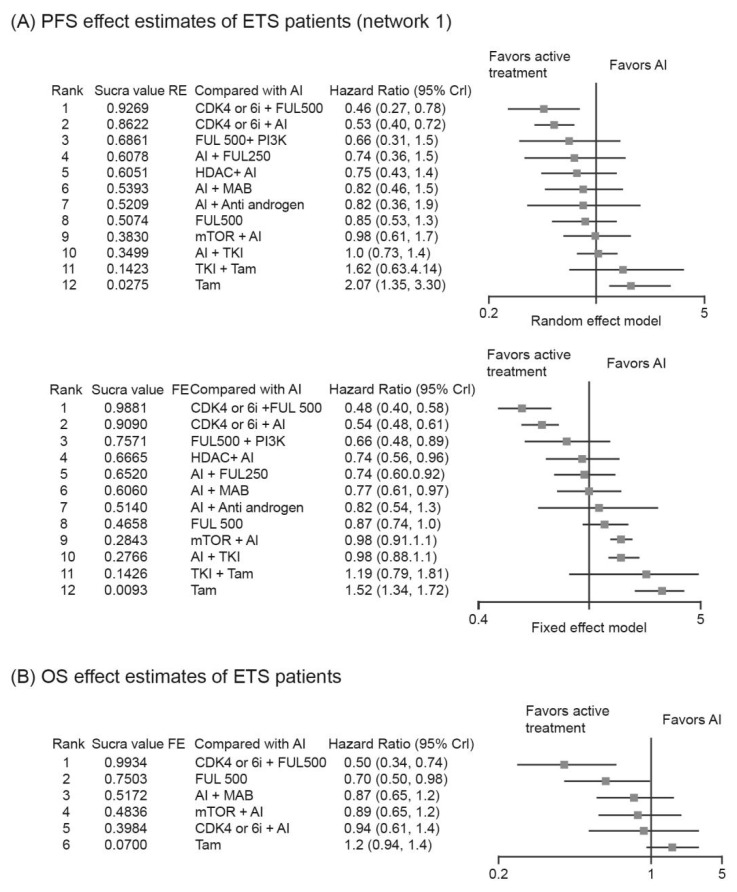
SUCRA values for treatment regimens and relative effect. (**A**) PFS effect estimates of ETS patients (network 1); (**B**) OS effect estimates of ETS patients (network 1).

A total of 34 trials were included for the PFS analysis including 13 different regimens for the major network and two regimens for the minor network. Among the 13 regimens, CDK4/6i + Fulvestrant (Ful 500, structural representation in Supplementary Figure S5) (RE: HR, 0.46; 95% CrI, 0.27–0.78; FE: HR, 0.48; 95% CrI, 0.40–0.58) was the best regimen and significantly better than aromatase inhibitor (AI), with a SUCRA value of 0.93 in the RE model and 0.99 in the FE model, followed by CDK4/6i + AI (RE: HR, 0.53; 95% CrI, 0.40–0.72; SUCRA, 0.86; FE: HR, 0.54; 95% CrI, 0.48–0.61; SUCRA, 0.91) and Ful 500 + PI3Ki (RE: HR, 0.66; 95% CrI, 0.48–0.89; SUCRA, 0.69; FE: HR, 0.66; 95% CrI, 0.48–0.89; SUCRA, 0.76) (Figure 2A and Figure 3A). The minor network revealed that Tyrosine kinase inhibitors (TKI) + Ful 500 was a better treatment regimen (HR, 0.88; 95% CrI, 0.45–1.7; SUCRA, 0.65) than Ful 250. 

A total of eight studies reported OS in patients with ETS and a network of seven regimens was constructed (Figure 3B). CDK4/6i + Ful 500 (HR, 0.50; 95% CrI, 0.34–0.74) ranked the highest, with a SUCRA value of 0.99, without statistical significance among the top three regimens (Figure 2B).

#### 3.3.1. Bone-Only and Visceral Metastasis

A total of eight studies reported PFS based on visceral metastasis and a network of seven regimens was constructed. The SUCRA value was highest for CDK4/6i + AI (HR, 0.56; 95% CrI, 0.44–0.70; SUCRA, 0.92), followed by CDK4/6i + Ful 500 (HR, 0.60; 95% CrI, 0.46–0.79; SUCRA, 0.78), Tam (HR, 0.66; 95% CrI, 0.54–0.80; SUCRA, 0.67) and AI + monoclonal antibody (MAB) (HR, 0.69; 95% CrI, 0.53–0.90; SUCRA, 0.62).

In patients with bone metastasis, among six treatment regimens reported in six studies, CDK4/6i + Ful 500 (HR, 0.43; 95% CrI, 0.24–0.76) was the most favorable with the highest SUCRA value of 0.90, followed by CDK4/6i + AI (HR, 0.44; 95% CrI, 0.28–0.69; SUCRA, 0.87) and Ful (HR, 0.71; 95% CrI, 0.34–1.5; SUCRA, 0.45).

#### 3.3.2. Relative Efficacy of Targeted-Based Regimens

Among the 10 studies reporting the efficacy of CDK4/6i-based regimens, a network with seven regimens was constructed. Sensitivity analysis revealed high risk of bias for the PALOMA-1/TRIO 18 [12] study and hence this was removed from the analysis. Abemaciclib (Abe) + AI (HR, 0.52; 95% CrI, 0.42–0.66) was the most effective regimen, with a SUCRA value of 0.94, followed by Palbociclib (Pal) + AI (HR, 0.64; 95% CrI, 0.53–0.78; SUCRA, 0.72), Ribociclib (Rib) + AI (HR, 0.65; 95% CrI, 0.47–0.89; SUCRA, 0.68), Pal + Ful (HR, 0.70; 95% CrI, 0.51–0.98; SUCRA, 0.59), Rib + Ful (HR, 0.87; 95% CrI, 0.54–1.4; SUCRA, 0.36) and Ful (HR, 1.6; 95% CrI, 1.1–2.3; SUCRA, 0.002) compared with AI with statistically significant differences observed among the top three regimens. 

A network of three regimens was constructed among two studies with HDACi. Tucidinostat (Tuc) + AI (HR, 0.68; 95% CrI, 0.50–0.93) demonstrated statistically significant efficacy, with a SUCRA value of 0.89, followed by entinostat (Ent) + AI (HR, 0.85; 95% CrI, 0.54–1.3; SUCRA, 0.49) and AI (SUCRA, 0.12).

Among the two studies with PI3Ki, a network of three regimens was constructed and, among them, buparlisib (Bup) + Ful (HR, 0.87; 95% CrI, 0.35–2.2) was the best, with a SUCRA value of 0.80, followed by alpelisib (Alp) + Ful (HR, 0.75; 95% CrI, 0.57–0.99; SUCRA, 0.50) compared with Ful (SUCRA, 0.20) with no statistically significant difference observed among the three regimens. 

### 3.4. Analysis of PFS/OS in Patients with ETR

**Figure 4:**
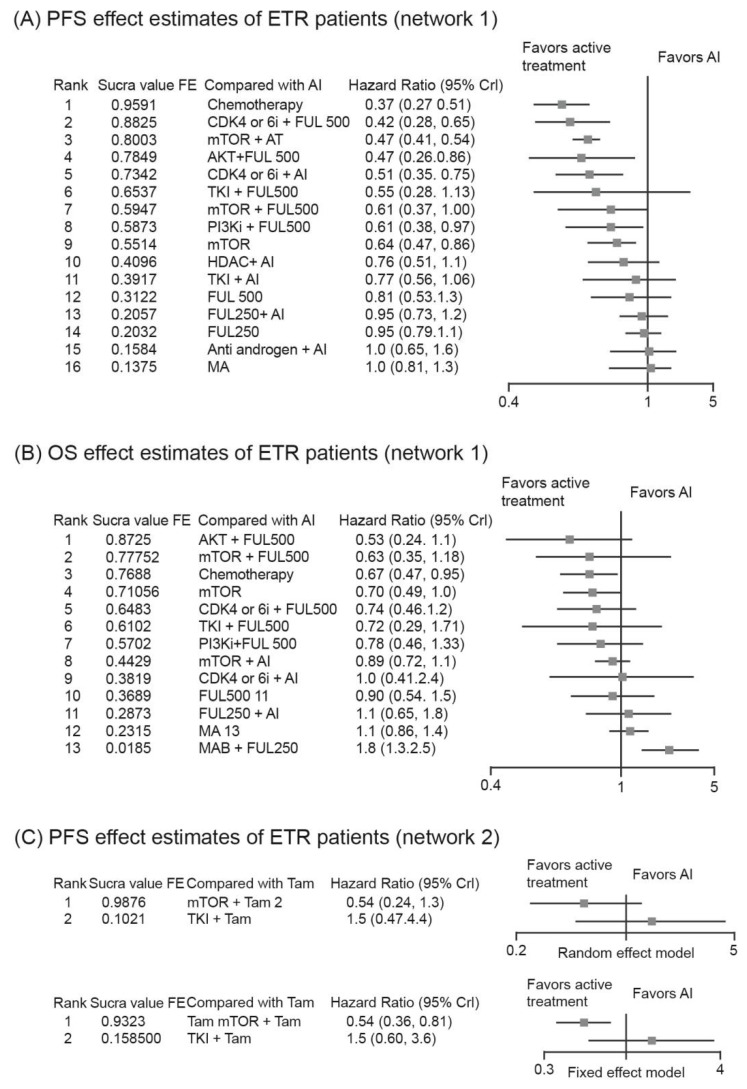
SUCRA values for treatment regimens and relative effect. (**A**) OS effect estimates of ETR patients; (**B**) OS effect estimates of ETR patients (network 1); (**C**) PFS effect estimates of ETR patients (network 2).

Out of the total studies, a major network with 31 studies including 17 regimens and a minor network with two studies and three regimens were constructed. Among the regimens considered in the major evidence network, chemotherapy (HR, 0.37; 95% CrI, 0.27–0.51) demonstrated statistically significant benefits, with a SUCRA value of 0.96, followed by CDK4/6i + Ful 500 (HR, 0.42; 95% CrI, 0.28–0.65; SUCRA, 0.88) and mTOR + AI (HR, 0.47; 95% CrI, 0.41–0.55; SUCRA, 0.80). The other regimens considered in the major network were provided in Figure 2C–E and Figure 4A,C. Three regimens were considered for the minor network, among which mTOR + tamoxifen (Tam) had a higher SUCRA (FE: 0.99, RE: 0.93), followed by Tam (FE: 0.41, RE: 0.41), with a statistically significant HR for mTOR + Tam vs. Tam (HR, 0.54; 95% CrI, 0.36–0.81) (Figure 4C).

The evaluation of OS included a total of 19 studies and two different evidence networks were constructed. Network 1 consisted of 14 regimens (Figure 2C andFigure 4B), out of which AKT + Ful 500 (HR, 0.53; 95% CrI, 0.24–1.1) ranked the highest, with a SUCRA value of 0.87, followed by mTOR + Ful500 (HR, 0.64; 95% CrI, 0.35–1.19; SUCRA, 0.78) and chemotherapy (HR, 0.67; 95% CrI, 0.47–0.95; SUCRA, 0.79), without statistically significant difference among them. In network 2, mTOR + Tam (HR, 0.45; 95% CrI, 0.14–1.4) was a better regimen than Tam with SUCRA value of 0.93. 

#### 3.4.1. Bone-Only and Visceral Metastasis

Among patients with visceral metastasis, five studies with three regimens were considered. CDK4/6i + Ful 500 was the most beneficial treatment regimen, with a SUCRA value of 0.99, followed by PI3Ki + Ful 500 (SUCRA, 0.49), with statistically significant improvement in PFS observed for CDK4/6i + Ful500 in comparison to Ful 500 (HR, 0.47; 95% CrI, 0.39–0.58) and PI3Ki + Ful 500 (HR, 0.76; 95% CrI, 0.64–0.90). The minor network with one study and two regimens revealed that mTOR was a better regimen than AI (HR, 0.47; 95% CrI, 0.37–0.60, SUCRA, 0.95)

Five studies reporting HRs stratified by patients with bone metastasis along with three regimens were included for the major network evidence analysis. CDK4/6i + Ful 500 (HR, 0.52; 95% CrI, 0.37–0.73) ranked the highest, with a SUCRA value of 0.94 followed by PI3Ki + Ful 500 (HR, 0.68; 95% CrI, 0.50–0.92; SUCRA, 0.56) in comparison with Ful 500 (SUCRA: 0.003) with significant differences observed. The minor network with one study and two regimens showed that mTOR + Ful was a better treatment than AI (HR, 0.33; 95% CrI, 0.078–1.4; SUCRA, 0.95).

#### 3.4.2. Relative Efficacy of Targeted-Based Regimens 

A total of six studies with six regimens reported the efficacy of the available CDK4/6i in combination with other drugs. Among the Ful-containing regimens, dalpiciclib + Ful 500 (HR, 0.42; 95% CrI, 0.31–0.57) was significantly better than Ful 500, with a SUCRA value of 0.90, followed by Abe + Ful 500 (HR, 0.52; 95% CrI, 0.43–0.62; SUCRA, 0.65). Among the three AI-containing regimens with three studies, Rib + AI (HR, 0.37; 95% CrI, 0.24–0.60; SUCRA, 0.93) was the best, followed by Pal + AI (HR, 0.37; 95% CrI, 0.24–0.60; SUCRA, 0.57), both significantly better than AI alone.

Among the two HDACi, Ent + AI (HR, 0.47; 95% CrI, 0.23–0.97) was the best, with a SUCRA value of 0.96, followed by Tuc + AI (HR, 0.92; 95% CrI, 0.58–1.5; SUCRA, 0.35) compared with AI (SUCRA, 0.19), but there was no statistically significant difference among the three regimens. 

A total of four studies compared the efficacy of the PI3Ki with four regimens, which revealed Bup + Ful (HR, 0.67, 95% CrI, 0.53–0.84) to be the most efficacious, with a SUCRA value of 0.76, followed by Alp + Ful (HR, 0.65; 95% CrI, 0.32–1.3; SUCRA, 0.71) and Pictilisib + Ful (HR, 0.80; 95% CrI, 0.58–1.1; SUCRA, 0.47), with Bup + Ful revealing significant improvement in PFS in comparison to Ful 500 (HR, 0.67; 95% CrI, 0.53–0.84).

#### 3.4.3. Menopausal Status

Thirty studies were included for the analysis of PFS among post-menopausal women with 17 regimens. Chemotherapy was found to be the most significantly better regimen than Ful 500 (HR, 0.45; 95% CrI, 0.33–0.61; SUCRA, 0.96), followed by CDK4/6i + Ful 500 (HR, 0.51; 95% CrI, 0.44–0.59; SUCRA, 0.89). Network 2 with two studies and three regimens showed that mTOR + Tam was significantly better than Tam (HR, 0.54; 95% CrI, 0.36–0.79; SUCRA, 0.99) and TKI + Tam (HR, 1.5; 95% CrI, 0.65–3.4; SUCRA, 0.09).

Two studies were included for the construction of network 1 for the pre-menopausal status. CDK4/6i + Ful 500 was better than Ful 500 (HR, 0.54; 95% CrI, 0.25–1.2; SUCRA, 0.96). Similarly, network 2 showed that mTOR + AI was better than AI (HR, 0.53; 95% CrI, 0.23–1.2; SUCRA, 0.94).

The hazards ratio and the respective 95% CrI are provided in online Supplementary Tables S2–S37.

## 4. Discussion

Over the past decade, several RCTs have led to the introduction of innovative targeted therapeutic strategies (mTOR, HDAC, CDK4/6, PI3K inhibitors, etc.) in combination with ETs into clinical practice. Furthermore, pivotal RCTs have proven the efficacy of these combinations as first and subsequent lines of treatment for HR+/HER2- mBC with substantial improvement in patient outcomes [1,2].

This network meta-analysis assessed the relative efficacies and provided a rank order of ET-based regimens in key endpoints PFS and OS for HR+/HER2- mBC patients with different endocrine sensitivity statuses. Based on the systematic review, the endocrine therapies were used in combination with the regimens targeting the CDK4/6, PI3K and HDAC pathways, which led to significant improvement in therapeutic outcomes. The main strength of this current network meta-analysis is the extensive analysis of all possible subgroups of patients and the number of studies included for the analysis. Given the geometry of the networks, the SUCRA values may guide clinical decision making by indicating ranking probabilities for each treatment.

The results of this study suggest that CDK4/6i in combination with Ful 500 is likely to be the best treatment option in terms of both PFS and OS benefit among ETS patients. The second-best options for treatment with significant differences for ETS patients were CDK4/6i + AI for improving PFS outcomes, and Ful 500 for improving OS outcomes. This was consistent with a recent network meta-analysis by Brandao et al. which reported CDK4/6i + Ful 500 to be the most effective treatment option for ETS patients [9]. Similar results were also reported in another network meta-analysis where addition of CDK4/6i to ET significantly improved median PFS (pooled HR = 0.547, *p* < 0.001) and OS (pooled HR = 0.755, *p* < 0.001). In addition, the PI3Ki + Ful therapy provided meaningful improvement in PFS (pooled HR = 0.686, *p* < 0.001), which was also noticed in our analysis [70].

In patients with ETR, chemotherapy has a high likelihood of therapeutic success, followed by CDK4/6i and AKTi in combination with fulvestrant. However, the network meta-analysis of 29 trials in ETR patients reported CDK4/6i + Ful 500 to be the best among 20 compared regimens, followed by capivasertib + Ful 500 as the second-best regimen [9]. However, in the current study, a total of 35 studies were included and all chemotherapeutic drugs were combined into a single drug class for comparison. Especially in cases of ETR-PFS and post-menopausal status in ETR patients containing chemotherapy arms, sensitivity analysis revealed a change in the treatment regimens after exclusion of Jeresaleum et al. [53] and Martin et al. [56]. The difference in the included trials and classification of regimens could be the reason for observed discordance. Nevertheless, the previous study also reported a lack of statistical significance among the top regimens [9].

Patients with visceral metastases have poor prognosis compared to those without visceral metastases among women with HR+/HER2- breast cancer. International guidelines recommend ET over chemotherapy as the frontline treatment option for mBC with HR+/HER2- type. Previous studies also revealed that treating patients with CDK4/6i + ET provided the advantage of PFS over ET alone in patients with or without visceral metastasis in both ETS and ETR settings [10]. Our study also revealed concordant findings, with CDK4/6i + FUL500 being the best regimen for ETS patients and CDK4/6i + Ful 500 for ETR patients. In patients with bone-only mBC, the treatment course is varied. However, bone-only disease is uncommon, and often observed in combination with other non-bone or non-visceral sites in clinical trials. A meta-analysis revealed that CDK4/6i + ET in combination improved PFS (HR, 0.54; 95% CI, 0.39–0.75; *p* < 0.001) for first-line treatment in bone-only disease settings, which is similar to our study findings [70].

We observed that chemotherapy was the best regimen for post-menopausal women with breast cancer. This is inconsistent with several published studies that reported hormone therapies with or without targeted therapies in post-menopausal women with HR+/HER2- mBC as the best treatment option. However, it must be noted that chemotherapy as an upfront treatment remains common even in the absence of visceral crisis and, hence, these results are to be interpreted with caution. This was also observed in a trial by Giuliano et al. [71] where chemotherapy or hormone therapy regimen was non-inferior to palbociclib plus letrozole for improving PFS. Though CDK4/6i + Ful500 was a better regimen in pre-menopausal women and consistent with previous studies [10,72], the number of studies were insufficient to derive to a meaningful conclusion. Combination of targeted therapies with ETs also revealed interesting results. In two of the meta-analyses, HDACi + ET was associated with prolonged PFS and OS compared to ET [73] or AI alone [74]. Accordingly, HDACi + ET was found to be a suitable treatment option in patients with HDAC modifications, with Tuc + AI and Ent + AI as the best regimen for patients with ETS and ETR status, respectively.

The classification of endocrine sensitivity statuses was as per the information provided in the included trials. However, if the trial did not categorically mention the endocrine status, then alternative information from the trial was used to determine this. For instance, the S0226 trial did not mention the endocrine status of the included patients in the initial publication with PFS data. Hence, patients with a previous history of tamoxifen administration were considered as endocrine-resistant patients [27]. However, in the subsequent publication with OS data, HR for patients stratified into endocrine-sensitive and -resistant were provided and the same were used for analysis [28]. 

### Limitations

Several limitations to this study must be acknowledged. Firstly, for subgroup analysis of bone-only metastasis, some of the studies provided only non-visceral metastasis, which was considered as bone-only metastasis for comparison. The MIRACLE trial [43], which evaluated the efficacy of everolimus in combination with AI in comparison to AI alone, provided HR stratified by visceral and non-visceral metastasis. The HR for non-visceral metastasis was considered as bone-only metastasis for analysis. Secondly, the effect estimates of the OS results may not be matured enough and may require a longer follow-up time for precise evaluation. In cases of patients with ETR, studies with fulvestrant and AI were distributed into two different networks and, hence, their relative efficacies with respect to extending the OS could not be ascertained. Thirdly, due to a large degree of heterogeneity across trials, network-based meta-analysis could not be performed for defined biomarker subgroups, such as patients with PIK3CA mutations.

## 5. Conclusions

Our comprehensive network meta-analysis suggests that CDK4/6i combination with ETs may be the best treatment option in terms of PFS for ETS patients and chemotherapy may be the best regimen for ETR patients with HR+/HER2- mBC. Moreover, CDK4/6i + ETs is likely to improve bone or visceral metastases irrespective of ETR or ETS status. Further matured OS data need to be assessed to conclude the OS benefit of the different ET-based treatment regimens. 

## Figures and Tables

**Table 1:** Baseline characteristics of the included trials.

S. No	Study Name	Treatment Regimen	Menopausal Status	Patients (N)	Phase	Centers	Previous CT	Endocrine Status s/r/^m^	1st ET
1	Ibrahim et al., 2011 [13]	SAI + MAB	Post	110	II	multi	no	s	no
		SAI							
2	Goetz et al., 2017 [15]	CDK4/6i + NSAI	Post	493	III	multi	no	s	yes
		NSAI							
3	Zhang et al., 2020 [16]	CDK4/6i + NSAI	Post	306	III	multi	no	s	no
		NSAI							
		CDK4/6i + FUL	Post	157	III	multi	no	r	yes
		FUL500							
4	Antonio C. Wolff et al., 2013 [17]	MTOR + NSAI	Post	1112	III	multi	no	s	no
		NSAI							
5	Dickler et al., 2016 [18]	NSAI + MAB	Post	343	II	multi	≤1 line	s	yes: ≤4 weeks
		NSAI							
6	Goss et al., 2007 [19]	SAI + TOR	Post	865	III	multi	yes	s	no
		SAI							
7	Cristofanilli et al., 2010 [20]	NSAI + TKI	Post	93	II	multi	no	s	no
		NSAI							
8	Krop et al., 2020 [21]	AI + anti-androgen	Post	127	II	multi	≤1 line	s	yes
		AI							
9	Albanell et al., 2020 [22]	CDK4/6i + Ful 500	Post	189	II	multi	no	s	no
		Ful 500							
10	Llombart-Cussac et al., 2021 [23]	CDK4/6i + Ful 500	Multi	486	II	muti	no	s	yes
		CDK4/6i + NSAI							
11	Mouridsen et al., 2001 [24]	NSAI	Post	907	III	multi	≤1 line	s	no
		Tam							
12	Paridaens et al., 2008 [25]	SAI	Post	371	III	multi	≤1 line	s	no
		Tam							
13	Milla-Santos et al., 2003 [23]	NSAI	Post	238	III	multi	no	s	no
		Tam							
14	Paul et al., 2019 [26]	AI + TKI	Post	120	II	multi	≤1 line	m	no
		AI							
15	Mehta et al., 2012 [27,28]	AI + Ful 250	Post	694	III	multi	yes	m	yes
		AI							
16	Finn et al., 2015, Finn et al., 2020 [29,30]	CDK4/6i + AI	Post	165	II	multi	no	m	no
		AI							
17	Finn et al., 2016 [31], Rugo et al., 2019 [32], Finn et al., 2017 [33]	CDK4/6i + AI	Post	666	III	multi	no	m	yes
		AI							
18	Cristofanilli et al., 2016 [34]	CDK4/6i + ful500	Both	521	III	multi	≤1 line	m	yes
		Ful 500							
19	Hortobagyi et al., 2018 [35]	CDK4/6i + AI	Post	668	III	multi	no	m	yes
		AI							
20	Slamon et al., 2021 [36], Slamon et al., 2018 [37], Slamon et al., 2020 [38], Hurvitz et al., [39]	CDK4/6i + ful500	Post	726	III	multi	no	m	yes
		Ful 500							
21	Johnston et al., 2013 [40]	AI + Ful 250	Post	723	III	multi	yes	m	yes
		Ful 250							
		Ful 250	Post	723	III	multi	yes	m	yes
		AI							
22	André et al., 2019 [41]	Ful500 + PI3K	Post	341	III	multi	yes	m	yes
		Ful 500							
23	Leo et al., 2018 [42]	Ful500 + PI3K	Post	432	III	multi	allowed	m	yes
		Ful 500							
24	Fan et al., 2021 [43]	NSAI + mTOR	Pre	199	II	multi	yes ≤1 line	m	yes: TAM
		NSAI							
25	Jiang et al., 2019 [44]	HDAC + SAI	Post	365	III	multi	≤1 line	m	yes
		SAI							
26	Yardley et al., 2011 [45]	HDAC + AI	Post	130	II	multi	≤1 line	m	yes: NSAI
		AI							
27	Xu et al., 2021 [46]	CDK4_or_6i_plus_AI	Post	340	III	multi	no	s	
		AI							
28	Treilleux et al., 2015 [47]	mTOR + TAM	Post	111	II	multi	≤1 line	r	yes: AI
		TAM							
29	Baselga et al., 2017 [48,49]	PI3Ki + Ful500	Post	1147	III	multi	≤1 line	m	yes
		FUL500							
30	Piccart et al., 2014 [50]	mTOR + AI	Post	724	III	multi	≤1 line	r	yes: AI
		AI							
31	Jones et al., 2020 [51]	AKT + FUL	Post	140	II	multi	≤1 line	r	yes: AI
		FUL500							
32	Krop et al., 2016 [52]	PI3Ki + Ful500	Post	168	II	multi	≤1 line	r	AI
		FUL500							
		PI3Ki + Ful500	Post	61	II	multi	≤1 line	r	AI
		FUL500							
33	Jerusalem et al., 2018 [53]	mTOR + AI	Post	309	II	multi	≤1 line	r	yes
		Mtor							
34	Schmid et al., 2019 [54]	mTOR + FUL500	Post	326	II	multi	≤1 line	r	yes
		FUL500							
35	Musolino et al., 2017 [55]	TKI + FUL500	Post	97	II	multi	no	r	yes
		FUL500							
36	Martin et al., 2021 [56]	CDK4/6i + FUL	Post	601	III	multi	≤1 line	r	yes: NSAI
		Chemotherapy							
37	Kornblum et al., 2018 [57]	mTOR + FUL500	Post	131	II	multi	≤1 line	r	yes: AI
		FUL500							
38	Xu et al., 2021 [58]	CDK4/6i + FUL	Both	361	III	multi	≤1 line	r	yes
		FUL500							
39	Buzdar et al., 2002 [59]	AI	Post	602	II	multi	≤1 line	r	yes
		MA							
40	Sledge et al., 2017 [60,61]	CDK4/6i + FUL	Both	669	III	multi	no	r	yes
		FUL500							
41	Shao et al., 2021 [62]	mTOR + AI	Post	159	Ⅱ	multi	yes	r	yes
		AI							
42	Johnston et al., 2009 [63]	TKI + AI	Post	1286	III	multi	no	m	yes
		AI							
43	Robertson et al., 2012 [64], Robertson et al., 2009 [65] Roberson et al., 2012 [64]	FUL500	Post	205	III	multi	yes	s	no
		AI							
44	Hyams et al., 2013 [66]	TKI + FUL250	Post	62	III	multi	≤1 line	s	yes
		FUL250							
45	Johnston et al., 2016 [67]	TKI + AI	Post	359	II	multi	≤1 line	s	yes
		AI							
46	Robertson et al., 2016 [68]	FUL	Post	462	III	multi	allowed	s	yes
		AI							
47	Osborne et al., 2002 [69]	FUL	Post	400	III	multi	no	m	yes
		AI							

## Supplementary Materials

The following supporting information can be downloaded at: https://www.mdpi.com/article/10.3390/cancers14246100/s1, Table S1. Electronic search strategy. Table S2. (A) PFS effect estimates of ETS patients for pairwise comparisons: hazard ratio (95% credibility interval, CrI) (network 1), (B) PFS effect estimates of ETS patients for pairwise comparisons: hazard ratio (95% credibility interval, CrI) (network 2), (C) OS effect estimates of ETS patients for pairwise comparisons: hazard ratio (95% credibility interval, CrI). Table S3. (A) PFS effect estimates of ETR patients for pairwise comparisons: hazard ratio (95% credibility interval, CrI) (network 1). (B) PFS effect estimates of ETR patients for pairwise comparisons: hazard ratio (95% credibility interval, CrI) (network 2), (C) OS effect estimates of ETR patients for pairwise comparisons: hazard ratio (95% credibility interval, CrI) (network 1), (D) OS effect estimates of ETR patients for pairwise comparisons: hazard ratio (95% credibility interval, CrI) (network 2). Table S4. SUCRA values of PFS for CDK4/6 inhibitors in ETS patients. Table S5. Effect estimates of PFS for CDK4/6 inhibitors in ETS patients: hazard ratio (95% credibility interval, CrI). Table S6. SUCRA values for PI3ki in ETS patients. Table S7. Effect estimates of PFS for PI3ki in ETS patients hazard ratio (95% credibility interval, CrI). Table S8. SUCRA values of PFS for HDACi in ETS patients. Table S9. Effect estimates of PFS for HDACi in ETS patients hazard ratio (95% credibility interval, CrI). Table S10. SUCRA of PFS for visceral metastases in ETS patients. Table S11. Effect estimates of PFS for visceral metastases in ETS patients: hazard ratio (95% credibility interval, CrI). Table S12. SUCRA values of PFS for bone metastases in ETS patients. Table S13. Effect estimates of PFS for bone metastases in ETS patients: hazard ratio (95% credibility interval, CrI). Table S14. SUCRA values of PFS for CDK4/6 inhibitors in ETR patients (Network 1). Table S15. Effect estimates of PFS for CDK4/6 inhibitors in ETR patients: hazard ratio (95% credibility interval, CrI) (Network 1). Table S16. SUCRA values of PFS for CDK4/6 inhibitors in ETR patients (Network 2). Table S17. Effect estimates of PFS for CDK4/6 inhibitors in ETR patients: hazard ratio (95% credibility interval, CrI) (Network 2). Table S18. SUCRA values of PFS for PI3ki in ETR patients. Table S19. Effect estimates of PFS for PI3ki in ETR patients: hazard ratio (95% credibility interval, CrI). Table S20. SUCRA values of PFS for HDACi in ETR patients. Table S21. Effect estimates of PFS for HDACi in ETR patients: hazard ratio (95% credibility interval, CrI). Table S22. SUCRA values of PFS for visceral metastases in ETR patients (network 1). Table S23. Effect estimates of PFS for visceral metastases in ETR patients: hazard ratio (95% credibility interval, CrI) (network 1). Table S24. SUCRA values of PFS for visceral metastases in ETR patients (network 2). Table S25. Effect estimates of PFS for visceral metastases in ETR patients: hazard ratio (95% credibility interval, CrI) (network 2). Table S26. SUCRA values of PFS for bone metastases in ETR patients (network 1). Table S27. Effect estimates of PFS for bone metastases in ETR patients: hazard ratio (95% credibility interval, CrI) (network 1). Table S28. SUCRA values of PFS for bone metastases in ETR patients (network 2). Table S29. Effect estimates of PFS for bone metastases in ETR patients: hazard ratio (95% credibility interval, CrI) (network 2). Table S30. SUCRA values of PFS for post-menopausal women in ETR patients (network 1). Table S31. Effect estimates of PFS for post-menopausal women in ETR patients: hazard ratio (95% credibility interval, CrI) (network 1). Table S32. SUCRA values of PFS for post-menopausal women in ETR patients (network 2). Table S33. Effect estimates of PFS for post-menopausal women in ETR patients: hazard ratio (95% credibility interval, CrI) (network 2). Table S34. SUCRA values of PFS for pre-menopausal women in ETR patients (network 1). Table S35. Effect estimates of PFS for pre-menopausal women in ETR patients: hazard ratio (95% credibility interval, CrI) (network 1). Table S36. SUCRA values of PFS for pre-menopausal women in ETR patients (network 2). Table S37. Effect estimates of PFS for pre-menopausal women in ETR patients: hazard ratio (95% credibility interval, CrI) (network 2). Figure S1. Risk of bias across studies (main analysis in ETS and ETR patients). Figure S2. Risk of bias across studies (subgroup analysis for targeted regimens in ETS patients). Figure S3. Risk of bias across studies (subgroup analysis for targeted regimens and menopausal women in ETR patients). Figure S4. Quality assessment data for individual studies. Figure S5. Chemical structure of fulvestrant.
