# Supplementary material for: Endocrine Therapy-Based Strategies for Metastatic Breast Cancer with Different Endocrine Sensitivity Statuses: A Systematic Review and Network Meta-Analysis
Source: Cancers (Basel). 2022 Dec 11;14(24):6100. doi: 10.3390/cancers14246100 (PMC9776369; doi:10.3390/cancers14246100)
Supplement: Supplementary file 1 [file cancers-14-06100-s001.zip › cancers-2049628-supplementary.pdf]

Supplementary Appendix

Endocrine Therapy-Based Strategies for Metastatic Breast Cancer with Different Endocrine Sensitivity Statuses：A Systematic Review and Network Meta-Analysis

eTable S1. Electronic search strategy

eTable S2A. PFS effect estimates of ETS patients for pairwise comparisons: hazard ratio (95% credibility interval, CrI) (network 1)

eTable S2B. PFS effect estimates of ETS patients for pairwise comparisons: hazard ratio (95% credibility interval, CrI) (network 2)

eTable S2C. OS effect estimates of ETS patients for pairwise comparisons: hazard ratio (95% credibility interval, CrI)

eTable S3A. PFS effect estimates of ETR patients for pairwise comparisons: hazard ratio (95% credibility interval, CrI) (network 1)

eTable S3B. PFS effect estimates of ETR patients for pairwise comparisons: hazard ratio (95% credibility interval, CrI) (network 2)

eTable S3C. OS effect estimates of ETR patients for pairwise comparisons: hazard ratio (95% credibility interval, CrI) (network 1)

eTable S3D. OS effect estimates of ETR patients for pairwise comparisons: hazard ratio (95% credibility interval, CrI) (network 2)

eTable S4. SUCRA values of PFS for CDK4/6 inhibitors in ETS patients

eTable S5. Effect estimates of PFS for CDK4/6 inhibitors in ETS patients：hazard ratio (95% credibility interval, CrI)

eTable S6. SUCRA values for PI3ki in ETS patients

eTable S7. Effect estimates of PFS for PI3ki in ETS patients hazard ratio (95% credibility interval, CrI)

eTable S8. SUCRA values of PFS for HDACi in ETS patients

eTable S9. Effect estimates of PFS for HDACi in ETS patients hazard ratio (95% credibility interval, CrI)

eTable S10. SUCRA of PFS for visceral metastases in ETS patients

eTable S11. Effect estimates of PFS for visceral metastases in ETS patients：hazard ratio (95% credibility interval, CrI)

eTable S12. SUCRA values of PFS for bone metastases in ETS patients

eTable S13. Effect estimates of PFS for bone metastases in ETS patients：hazard ratio (95% credibility interval, CrI)

eTable S14. SUCRA values of PFS for CDK4/6 inhibitors in ETR patients (Network 1)

eTable S15. Effect estimates of PFS for CDK4/6 inhibitors in ETR patients：hazard ratio (95% credibility interval, CrI) (Network 1)

eTable S16. SUCRA values of PFS for CDK4/6 inhibitors in ETR patients (Network 2)

eTable S17. Effect estimates of PFS for CDK4/6 inhibitors in ETR patients：hazard ratio (95% credibility interval, CrI) (Network 2)

eTable S18. SUCRA values of PFS for PI3ki in ETR patients

eTable S19. Effect estimates of PFS for PI3ki in ETR patients：hazard ratio (95% credibility interval, CrI)

eTable S20. SUCRA values of PFS for HDACi in ETR patients

eTable S21. Effect estimates of PFS for HDACi in ETR patients：hazard ratio (95% credibility interval, CrI)

eTable S22. SUCRA values of PFS for visceral metastases in ETR patients (network 1)

eTable S23. Effect estimates of PFS for visceral metastases in ETR patients：hazard ratio (95% credibility interval, CrI) (network 1)

eTable S24. SUCRA values of PFS for visceral metastases in ETR patients (network 2)

eTable S25. Effect estimates of PFS for visceral metastases in ETR patients：hazard ratio (95% credibility interval, CrI) (network 2)

eTable S26. SUCRA values of PFS for bone metastases in ETR patients (network 1)

eTable S27. Effect estimates of PFS for bone metastases in ETR patients：hazard ratio (95% credibility interval, CrI) (network 1)

eTable S28. SUCRA values of PFS for bone metastases in ETR patients (network 2)

eTable S29. Effect estimates of PFS for bone metastases in ETR patients：hazard ratio (95% credibility interval, CrI) (network 2)

eTable S30. SUCRA values of PFS for post-menopausal women in ETR patients (network 1)

eTable S31. Effect estimates of PFS for post-menopausal women in ETR patients：hazard ratio (95% credibility interval, CrI) (network 1)

eTable S32. SUCRA values of PFS for post-menopausal women in ETR patients (network 2)

eTable S33. Effect estimates of PFS for post-menopausal women in ETR patients：hazard ratio (95% credibility interval, CrI) (network 2)

eTable S34. SUCRA values of PFS for pre-menopausal women in ETR patients (network 1)

eTable S35. Effect estimates of PFS for pre-menopausal women in ETR patients：hazard ratio (95% credibility interval, CrI) (network 1)

eTable S36. SUCRA values of PFS for pre-menopausal women in ETR patients (network 2)

eTable S37. Effect estimates of PFS for pre-menopausal women in ETR patients：hazard ratio (95% credibility interval, CrI) (network 2)

eFigure S1. Risk of bias across studies (main analysis in ETS and ETR patients)

eFigure S2. Risk of bias across studies (subgroup analysis for targeted regimens in ETS patients)

eFigure S3. Risk of bias across studies (subgroup analysis for targeted regimens and menopausal women in ETR patients)

eFigure S4. Quality assessment data for individual studies

eFigure S5. Chemical structure of fulvestrant

eTable S1. Electronic search strategy

#	**Searches**
1	trastuzumab/
2	trastuzumab.tw.
3	1 or 2
4	HER2-positive
5	advanced breast cancer/
6	advanced breast cancer.tw.
7	metastatic breast cancer/
8	metastatic breast cancer.tw.
9	locally advanced breast cancer/
10	locally advanced breast cancer.tw

11	6 or 7 or 8 or 9 or 10 or 11
12	disease progression/
13	disease progression.tw.
14	12 or 13
15	chemotherapy/
16	chemotherapy.tw.
17	15 or 16
18	3 and 4 and 12 and 14 and 17
19	randomized controlled trials/
20	randomized-controlled-trial.pt
21	controlled-clinical-trial.pt
22	random allocation/
23	19 or 20 or 21 or 22
24	research design/

25	comparative study
26	exp evaluation studies/
27	follow-up studies/
28	prospective studies/
29	25 or 26 or 27 or 28
30	23 or 24 or 29
31	28 and 30

eTable S2A. PFS effect estimates of ETS patients for pairwise comparisons: hazard ratio (95% credibility interval, CrI) (network 1)

Random effect model

**AI**	**0.8301 (0.3627, 1.853)**	0.7354 (0.3467, 1.525)	0.8123 (0.4648, 1.5)	1.005 (0.7383, 1.356)	0.5299 (0.3916, 0.7081)	0.4554 (0.2649, 0.769)	0.839 (0.5248, 1.325)	0.6615 (0.3112, 1.404)	0.752 (0.4274, 1.322)	2.073 (1.35, 3.302)	1.622 (0.6357, 4.145)
1.205 (0.5397, 2.757)	**AI_plus_anti_androgen**	0.8882 (0.2991, 2.575)	0.9849 (0.364, 2.728)	1.216 (0.5102, 2.836)	0.6427 (0.2593, 1.494)	0.5513 (0.2088, 1.45)	1.014 (0.3968, 2.586)	0.7962 (0.2635, 2.439)	0.9068 (0.3534, 2.478)	2.526 (1.033, 6.328)	1.976 (0.5757, 6.548)
1.36 (0.6557, 2.885)	1.126 (0.3883, 3.343)	**AI_plus_FUL250**	1.102 (0.437, 2.879)	1.374 (0.6109, 3.067)	0.7185 (0.332, 1.604)	0.6149 (0.2582, 1.573)	1.139 (0.4777, 2.841)	0.8992 (0.3035, 2.581)	1.018 (0.4245, 2.557)	2.815 (1.217, 6.841)	2.225 (0.6943, 7.331)
1.231 (0.6667, 2.151)	1.015 (0.3666, 2.747)	0.9076 (0.3473, 2.288)	**AI_plus_MAB**	1.229 (0.625, 2.345)	0.6485 (0.3297, 1.236)	0.5581 (0.248, 1.202)	1.033 (0.4674, 2.094)	0.8104 (0.3061, 2.099)	0.9288 (0.4034, 1.988)	2.554 (1.217, 5.361)	2.018 (0.6564, 5.939)
0.9954 (0.7377, 1.354)	0.8224 (0.3527, 1.96)	0.7277 (0.3261, 1.637)	0.8135 (0.4264, 1.6)	**AI_plus_TKI**	0.5279 (0.3451, 0.8095)	0.4512 (0.2463, 0.8375)	0.8337 (0.476, 1.453)	0.6609 (0.2844, 1.473)	0.7511 (0.3935, 1.412)	2.064 (1.225, 3.569)	1.628 (0.6059, 4.302)
1.887 (1.412, 2.554)	1.556 (0.6695, 3.856)	1.392 (0.6233, 3.012)	1.542 (0.8087, 3.033)	1.894 (1.235, 2.898)	**CDK4_or_6i_plus_AI**	0.8618 (0.5073, 1.453)	1.577 (0.947, 2.623)	1.243 (0.5728, 2.697)	1.423 (0.7485, 2.661)	3.911 (2.307, 6.917)	3.07 (1.123, 8.244)
2.196 (1.3, 3.774)	1.814 (0.6896, 4.79)	1.626 (0.6359, 3.873)	1.792 (0.832, 4.032)	2.216 (1.194, 4.06)	1.16 (0.6884, 1.971)	**CDK4_or_6i_plus_FUL500**	1.845 (1.247, 2.742)	1.439 (0.6932, 2.963)	1.659 (0.786, 3.562)	4.58 (2.331, 9.604)	3.592 (1.244, 10.54)
1.192 (0.7544, 1.905)	0.9866 (0.3867, 2.52)	0.8778 (0.3519, 2.093)	0.9682 (0.4774, 2.14)	1.199 (0.6883, 2.101)	0.6339 (0.3812, 1.056)	0.5421 (0.3646, 0.8022)	**FUL500**	0.7874 (0.4252, 1.442)	0.8979 (0.4364, 1.852)	2.464 (1.31, 4.928)	1.937 (0.7013, 5.563)
1.512 (0.7122, 3.213)	1.256 (0.4101, 3.795)	1.112 (0.3874, 3.295)	1.234 (0.4765, 3.267)	1.513 (0.6789, 3.516)	0.8047 (0.3709, 1.746)	0.6952 (0.3375, 1.443)	1.27 (0.6935, 2.352)	**Ful500_plus_PI3K**	1.141 (0.4475, 2.937)	3.165 (1.333, 7.749)	2.494 (0.7712, 8.42)
1.33 (0.7564, 2.34)	1.103 (0.4036, 2.829)	0.9823 (0.3911, 2.356)	1.077 (0.503, 2.479)	1.331 (0.7084, 2.541)	0.7027 (0.3758, 1.336)	0.6028 (0.2807, 1.272)	1.114 (0.54, 2.291)	0.8761 (0.3405, 2.235)	**HDAC_plus_AI**	2.76 (1.396, 5.578)	2.176 (0.7165, 6.34)
1.013 (0.6281, 1.621)	0.8311 (0.3199, 2.163)	0.7431 (0.3011, 1.775)	0.8183 (0.3978, 1.773)	1.018 (0.568, 1.782)	0.5359 (0.3015, 0.9255)	0.4602 (0.2191, 0.9113)	0.8504 (0.434, 1.627)	0.671 (0.2662, 1.6)	0.7633 (0.3585, 1.582)	2.098 (1.127, 4.007)	1.66 (0.5618, 4.735)

0.4823 (0.3028, 0.7406)	0.3959 (0.158, 0.9679)	0.3552 (0.1462, 0.8218)	0.3916 (0.1865, 0.8214)	0.4845 (0.2802, 0.8162)	0.2557 (0.1446, 0.4335)	0.2183 (0.1041, 0.429)	0.4059 (0.2029, 0.7636)	0.316 (0.1291, 0.7501)	0.3623 (0.1793, 0.7165)	**Tam**	0.7869 (0.3421, 1.731)
0.6166 (0.2412, 1.573)	0.5061 (0.1527, 1.737)	0.4494 (0.1364, 1.44)	0.4955 (0.1684, 1.524)	0.6142 (0.2325, 1.65)	0.3257 (0.1213, 0.8904)	0.2784 (0.09487, 0.8041)	0.5162 (0.1798, 1.426)	0.401 (0.1188, 1.297)	0.4596 (0.1577, 1.396)	1.271 (0.5776, 2.924)	**TKI_plus_Tam**

Fixed effect model

**AI**	0.8205 (0.5407, 1.244)	0.7396 (0.5946, 0.9213)	0.7765 (0.6142, 0.9767)	0.9816 (0.8831, 1.095)	0.5435 (0.483, 0.614)	0.4814 (0.398, 0.5844)	0.8675 (0.7366, 1.024)	0.6605 (0.4864, 0.8965)	0.7291 (0.5661, 0.9437)	1.529 (1.348, 1.727)	1.195 (0.7901, 1.809)
1.219 (0.804, 1.849)	**AI_plus_anti_androgen**	0.8993 (0.566, 1.446)	0.9504 (0.5895, 1.528)	1.198 (0.7792, 1.867)	0.6622 (0.432, 1.026)	0.5873 (0.3708, 0.9269)	1.058 (0.6758, 1.644)	0.8063 (0.4837, 1.347)	0.8891 (0.5459, 1.479)	1.863 (1.207, 2.899)	1.455 (0.8184, 2.644)
1.352 (1.085, 1.682)	1.112 (0.6915, 1.767)	**AI_plus_FUL250**	1.051 (0.7588, 1.446)	1.33 (1.036, 1.703)	0.7352 (0.5711, 0.949)	0.6512 (0.4844, 0.8786)	1.173 (0.8889, 1.55)	0.8913 (0.6138, 1.308)	0.9889 (0.704, 1.38)	2.068 (1.597, 2.677)	1.617 (0.9934, 2.619)
1.288 (1.024, 1.628)	1.052 (0.6544, 1.696)	0.9512 (0.6913, 1.318)	**AI_plus_MAB**	1.267 (0.9868, 1.633)	0.7033 (0.5402, 0.9097)	0.6199 (0.4625, 0.8378)	1.118 (0.8512, 1.474)	0.8538 (0.5817, 1.243)	0.9371 (0.6719, 1.32)	1.973 (1.519, 2.562)	1.54 (0.9618, 2.463)
1.019 (0.9132, 1.132)	0.8346 (0.5355, 1.283)	0.7517 (0.5871, 0.9652)	0.7891 (0.6123, 1.013)	**AI_plus_TKI**	0.5533 (0.4711, 0.6503)	0.4906 (0.3937, 0.6124)	0.8826 (0.7253, 1.072)	0.6728 (0.49, 0.9329)	0.7446 (0.5657, 0.9779)	1.558 (1.323, 1.839)	1.217 (0.7844, 1.878)
1.84 (1.629, 2.071)	1.51 (0.9745, 2.315)	1.36 (1.054, 1.751)	1.422 (1.099, 1.851)	1.807 (1.538, 2.123)	**CDK4_or_6i_plus_AI**	0.8846 (0.7294, 1.07)	1.595 (1.324, 1.914)	1.214 (0.8909, 1.666)	1.342 (1.011, 1.769)	2.809 (2.357, 3.338)	2.195 (1.427, 3.357)
2.077 (1.711, 2.513)	1.703 (1.079, 2.697)	1.536 (1.138, 2.064)	1.613 (1.194, 2.162)	2.038 (1.633, 2.54)	1.13 (0.9348, 1.371)	**CDK4_or_6i_plus_FUL500**	1.801 (1.538, 2.105)	1.371 (0.9989, 1.851)	1.515 (1.101, 2.077)	3.173 (2.514, 3.978)	2.487 (1.571, 3.933)
1.153 (0.9767, 1.358)	0.945 (0.6085, 1.48)	0.8527 (0.6451, 1.125)	0.8944 (0.6785, 1.175)	1.133 (0.9333, 1.379)	0.6268 (0.5226, 0.7555)	0.5552 (0.4752, 0.6503)	**FUL500**	0.7631 (0.5842, 0.9961)	0.8423 (0.6252, 1.138)	1.766 (1.426, 2.156)	1.38 (0.8795, 2.153)
1.514 (1.115, 2.056)	1.24 (0.7426, 2.068)	1.122 (0.7645, 1.629)	1.171 (0.8045, 1.719)	1.486 (1.072, 2.041)	0.8234 (0.6004, 1.122)	0.7296 (0.5403, 1.001)	1.31 (1.004, 1.712)	**Ful500_plus_PI3K**	1.106 (0.7464, 1.643)	2.316 (1.653, 3.188)	1.803 (1.082, 3.034)

1.372 (1.06, 1.766)	1.125 (0.6763, 1.832)	1.011 (0.7248, 1.42)	1.067 (0.7577, 1.488)	1.343 (1.023, 1.768)	0.7454 (0.5653, 0.9886)	0.6599 (0.4815, 0.9081)	1.187 (0.8789, 1.599)	0.9043 (0.6088, 1.34)	**HDAC_plus_AI**	2.096 (1.588, 2.792)	1.633 (1.004, 2.669)
1.02 (0.9451, 1.102)	0.8361 (0.5456, 1.288)	0.7538 (0.5938, 0.9504)	0.79 (0.6215, 1.005)	0.9993 (0.8792, 1.148)	0.5544 (0.4813, 0.637)	0.4901 (0.4007, 0.6038)	0.8841 (0.7422, 1.062)	0.6741 (0.4908, 0.9302)	0.7457 (0.5689, 0.9672)	1.557 (1.346, 1.799)	1.219 (0.7988, 1.86)
0.6539 (0.579, 0.7416)	0.5367 (0.345, 0.8285)	0.4836 (0.3735, 0.6263)	0.5069 (0.3903, 0.6581)	0.6417 (0.5438, 0.756)	0.356 (0.2995, 0.4243)	0.3152 (0.2514, 0.3977)	0.5663 (0.4639, 0.7012)	0.4318 (0.3137, 0.6048)	0.477 (0.3581, 0.6298)	**Tam**	0.7778 (0.5224, 1.177)
0.8367 (0.5527, 1.266)	0.6875 (0.3782, 1.222)	0.6184 (0.3819, 1.007)	0.6494 (0.4061, 1.04)	0.822 (0.5326, 1.275)	0.4556 (0.2979, 0.7008)	0.4021 (0.2542, 0.6364)	0.7247 (0.4644, 1.137)	0.5546 (0.3296, 0.9244)	0.6125 (0.3747, 0.9962)	1.286 (0.8494, 1.914)	**TKI_plus_Tam**

eTable S2B. PFS effect estimates of ETS patients for pairwise comparisons: hazard ratio (95% credibility interval, CrI) (network 2)

FUL250	0.9024 (0.4463, 1.751)
1.108 (0.571, 2.241)	TKI_plus_FUL250

eTable S2C. OS effect estimates of ETS patients for pairwise comparisons: hazard ratio (95% credibility interval, CrI)

**AI**	0.8699 (0.4467, 1.683)	0.9414 (0.4559, 1.942)	0.4949 (0.2198, 1.091)	0.6981 (0.3675, 1.362)	0.8907 (0.4603, 1.778)	1.163 (0.7625, 1.874)
1.15 (0.594, 2.239)	**AI_plus_MAB**	1.08 (0.3944, 2.823)	0.5682 (0.1956, 1.561)	0.8085 (0.3092, 1.998)	1.029 (0.4202, 2.639)	1.341 (0.5955, 2.96)
1.062 (0.5149, 2.193)	0.9258 (0.3542, 2.536)	**CDK4_or_6i_plus_AI**	0.5225 (0.1764, 1.481)	0.7457 (0.2839, 1.908)	0.9519 (0.3563, 2.536)	1.239 (0.5288, 2.922)
2.021 (0.9168, 4.551)	1.76 (0.6407, 5.113)	1.914 (0.6754, 5.669)	**CDK4_or_6i_plus_FUL500**	1.422 (0.9003, 2.283)	1.8 (0.6627, 5.345)	2.364 (0.9377, 6.019)
1.433 (0.7341, 2.721)	1.237 (0.5004, 3.234)	1.341 (0.524, 3.523)	0.7033 (0.438, 1.111)	**FUL500**	1.262 (0.5017, 3.393)	1.678 (0.753, 3.716)
1.123 (0.5626, 2.173)	0.9716 (0.3789, 2.38)	1.051 (0.3942, 2.807)	0.5555 (0.1871, 1.509)	0.7926 (0.2947, 1.993)	**mTOR_plus_AI**	1.305 (0.586, 2.962)
0.8598 (0.5335, 1.311)	0.7457 (0.3378, 1.679)	0.8069 (0.3422, 1.891)	0.423 (0.1661, 1.066)	0.5959 (0.2691, 1.328)	0.7664 (0.3376, 1.707)	**Tam**

eTable S3A. PFS effect estimates of ETR patients for pairwise comparisons: hazard ratio (95% credibility interval, CrI) (network 1)

**AI**	0.4795 (0.2674, 0.8839)	1.015 (0.6481, 1.588)	0.5142 (0.3539, 0.7414)	0.4259 (0.2788, 0.6538)	0.3763 (0.2757, 0.5173)	0.9537 (0.7985, 1.145)	0.9517 (0.7317, 1.239)	0.8192 (0.5301, 1.28)	0.7602 (0.5179, 1.118)	1.008 (0.8105, 1.253)	0.6408 (0.4736, 0.8661)	0.4747 (0.411, 0.5486)	0.606 (0.3776, 1.001)	0.6065 (0.3887, 0.9694)	0.7779 (0.5639, 1.069)	0.5595 (0.2889, 1.13)
2.086 (1.131, 3.74)	**AKT_plus_FUL500**	2.138 (1.003, 4.53)	1.079 (0.5295, 2.127)	0.8887 (0.5948, 1.353)	0.7844 (0.4861, 1.294)	1.993 (1.056, 3.694)	1.996 (1.034, 3.833)	1.712 (1.165, 2.547)	1.598 (0.7612, 3.207)	2.114 (1.101, 3.952)	1.351 (0.6987, 2.466)	0.9871 (0.5516, 1.738)	1.265 (0.8217, 1.949)	1.267 (0.8517, 1.923)	1.628 (0.8203, 3.141)	1.172 (0.6162, 2.248)
0.9852 (0.6299, 1.543)	0.4677 (0.2207, 0.9971)	**Anti_androgen_plus_AI**	0.5043 (0.2861, 0.8956)	0.4188 (0.2278, 0.7675)	0.3708 (0.2124, 0.6474)	0.9393 (0.5769, 1.514)	0.9418 (0.5581, 1.583)	0.8092 (0.4315, 1.512)	0.754 (0.4168, 1.31)	0.9946 (0.6066, 1.649)	0.6312 (0.3719, 1.099)	0.4669 (0.2915, 0.7485)	0.595 (0.3137, 1.145)	0.5984 (0.3158, 1.135)	0.7654 (0.4428, 1.332)	0.5539 (0.2421, 1.267)
1.945 (1.349, 2.825)	0.9265 (0.4702, 1.889)	1.983 (1.117, 3.496)	**CDK4_or_6i_plus_AI**	0.8275 (0.4743, 1.47)	0.7348 (0.4521, 1.192)	1.857 (1.238, 2.805)	1.86 (1.179, 2.902)	1.596 (0.9052, 2.857)	1.479 (0.8649, 2.53)	1.962 (1.277, 3.031)	1.243 (0.7739, 2.025)	0.9238 (0.6166, 1.375)	1.173 (0.6441, 2.182)	1.184 (0.6614, 2.122)	1.512 (0.9396, 2.499)	1.09 (0.5072, 2.397)
2.348 (1.529, 3.587)	1.125 (0.7391, 1.681)	2.388 (1.303, 4.39)	1.208 (0.6801, 2.108)	**CDK4_or_6i_plus_FUL500**	0.8836 (0.6663, 1.163)	2.236 (1.402, 3.569)	2.232 (1.365, 3.665)	1.927 (1.691, 2.2)	1.782 (0.9907, 3.186)	2.367 (1.463, 3.777)	1.505 (0.9401, 2.386)	1.116 (0.737, 1.645)	1.426 (1.137, 1.786)	1.425 (1.21, 1.7)	1.828 (1.079, 3.118)	1.316 (0.7796, 2.24)
2.657 (1.933, 3.628)	1.275 (0.7726, 2.057)	2.697 (1.545, 4.708)	1.361 (0.8391, 2.212)	1.132 (0.8598, 1.501)	**Chemotherapy**	2.542 (1.751, 3.659)	2.526 (1.701, 3.788)	2.183 (1.604, 2.978)	2.022 (1.22, 3.307)	2.687 (1.815, 3.932)	1.707 (1.155, 2.478)	1.259 (0.9453, 1.654)	1.614 (1.115, 2.322)	1.613 (1.171, 2.235)	2.07 (1.329, 3.21)	1.48 (0.8288, 2.716)
1.049 (0.8736, 1.252)	0.5019 (0.2707, 0.9473)	1.065 (0.6607, 1.733)	0.5385 (0.3565, 0.8077)	0.4472 (0.2802, 0.7135)	0.3934 (0.2733, 0.5712)	**FUL250**	0.9983 (0.828, 1.196)	0.8618 (0.5305, 1.4)	0.7964 (0.5253, 1.219)	1.058 (0.794, 1.411)	0.6739 (0.4696, 0.9562)	0.498 (0.3915, 0.6277)	0.6376 (0.3824, 1.07)	0.6379 (0.391, 1.035)	0.8168 (0.5665, 1.179)	0.584 (0.2936, 1.216)
1.051 (0.807, 1.367)	0.5009 (0.2609, 0.9672)	1.062 (0.6318, 1.792)	0.5375 (0.3446, 0.8481)	0.4481 (0.2728, 0.7327)	0.3959 (0.264, 0.5879)	1.002 (0.8361, 1.208)	**FUL250_plus_AI**	0.8661 (0.5147, 1.431)	0.8016 (0.5027, 1.251)	1.056 (0.7452, 1.491)	0.6726 (0.4473, 1.008)	0.4966 (0.3705, 0.6745)	0.6397 (0.3756, 1.1)	0.6394 (0.3794, 1.061)	0.8165 (0.546, 1.249)	0.5911 (0.2847, 1.237)

1.221 (0.7811, 1.886)	0.5843 (0.3927, 0.8585)	1.236 (0.6614, 2.318)	0.6264 (0.35, 1.105)	0.5189 (0.4546, 0.5913)	0.458 (0.3357, 0.6235)	1.16 (0.7144, 1.885)	1.155 (0.6986, 1.943)	**FUL500**	0.9289 (0.5052, 1.674)	1.23 (0.7412, 1.992)	0.7803 (0.4714, 1.259)	0.5772 (0.3715, 0.867)	0.7382 (0.6105, 0.888)	0.7388 (0.6683, 0.8201)	0.9457 (0.5508, 1.631)	0.6818 (0.4088, 1.153)
1.315 (0.8943, 1.931)	0.6257 (0.3119, 1.314)	1.326 (0.7635, 2.399)	0.676 (0.3952, 1.156)	0.5613 (0.3139, 1.009)	0.4944 (0.3024, 0.8196)	1.256 (0.8201, 1.904)	1.247 (0.7992, 1.989)	1.077 (0.5975, 1.98)	**HDAC_plus_AI**	1.321 (0.8561, 2.076)	0.845 (0.5126, 1.381)	0.622 (0.4116, 0.9423)	0.7947 (0.4272, 1.503)	0.7971 (0.4384, 1.457)	1.022 (0.6236, 1.688)	0.7382 (0.3395, 1.656)
0.9917 (0.7978, 1.234)	0.4731 (0.253, 0.9084)	1.005 (0.6063, 1.648)	0.5096 (0.3299, 0.7832)	0.4225 (0.2648, 0.6835)	0.3722 (0.2543, 0.5509)	0.9447 (0.7087, 1.259)	0.9474 (0.6705, 1.342)	0.813 (0.502, 1.349)	0.7571 (0.4817, 1.168)	**MA**	0.6368 (0.4364, 0.9209)	0.4705 (0.3606, 0.6144)	0.6005 (0.3584, 1.027)	0.6019 (0.3665, 1.008)	0.7734 (0.5193, 1.143)	0.5535 (0.2727, 1.15)
1.561 (1.155, 2.111)	0.7404 (0.4055, 1.431)	1.584 (0.9097, 2.689)	0.8045 (0.4938, 1.292)	0.6644 (0.4192, 1.064)	0.5857 (0.4036, 0.8657)	1.484 (1.046, 2.129)	1.487 (0.992, 2.235)	1.282 (0.7946, 2.122)	1.183 (0.7243, 1.951)	1.57 (1.086, 2.291)	**mTOR**	0.7405 (0.5671, 0.9596)	0.9459 (0.5674, 1.608)	0.9495 (0.5789, 1.572)	1.217 (0.781, 1.879)	0.8724 (0.4348, 1.796)
2.106 (1.823, 2.433)	1.013 (0.5754, 1.813)	2.142 (1.336, 3.431)	1.082 (0.7271, 1.622)	0.8963 (0.6079, 1.357)	0.7942 (0.6045, 1.058)	2.008 (1.593, 2.554)	2.014 (1.482, 2.699)	1.732 (1.153, 2.691)	1.608 (1.061, 2.429)	2.126 (1.628, 2.774)	1.35 (1.042, 1.763)	**mTOR_plus_AI**	1.277 (0.8146, 2.048)	1.283 (0.8415, 1.996)	1.64 (1.16, 2.309)	1.181 (0.6151, 2.343)
1.65 (0.999, 2.648)	0.7907 (0.513, 1.217)	1.681 (0.8733, 3.188)	0.8523 (0.4582, 1.553)	0.701 (0.5599, 0.8793)	0.6195 (0.4306, 0.8966)	1.568 (0.9345, 2.615)	1.563 (0.909, 2.662)	1.355 (1.126, 1.638)	1.258 (0.6654, 2.341)	1.665 (0.9734, 2.79)	1.057 (0.622, 1.762)	0.7829 (0.4882, 1.228)	**mTOR_plus_FUL500**	1.002 (0.8098, 1.241)	1.287 (0.7177, 2.279)	0.9258 (0.5351, 1.612)
1.649 (1.032, 2.573)	0.7894 (0.5201, 1.174)	1.671 (0.8809, 3.166)	0.8447 (0.4712, 1.512)	0.7015 (0.5881, 0.8268)	0.6201 (0.4474, 0.854)	1.568 (0.9662, 2.558)	1.564 (0.9422, 2.636)	1.354 (1.219, 1.496)	1.255 (0.6864, 2.281)	1.661 (0.9919, 2.729)	1.053 (0.6363, 1.728)	0.7797 (0.5009, 1.188)	0.998 (0.8056, 1.235)	**PI3Ki_plus_FUL500**	1.277 (0.7411, 2.24)	0.9213 (0.5445, 1.598)
1.285 (0.9355, 1.773)	0.6144 (0.3184, 1.219)	1.307 (0.751, 2.258)	0.6615 (0.4001, 1.064)	0.5472 (0.3207, 0.927)	0.4831 (0.3115, 0.7527)	1.224 (0.8481, 1.765)	1.225 (0.8007, 1.831)	1.057 (0.6131, 1.815)	0.9784 (0.5925, 1.604)	1.293 (0.8748, 1.926)	0.8214 (0.5323, 1.28)	0.6099 (0.4331, 0.8621)	0.777 (0.4388, 1.393)	0.7831 (0.4463, 1.349)	**TKI_plus_AI**	0.716 (0.3419, 1.545)

1.787 (0.8848, 3.461)	0.8529 (0.4448, 1.623)	1.805 (0.7893, 4.131)	0.9177 (0.4172, 1.972)	0.7598 (0.4465, 1.283)	0.6756 (0.3682, 1.206)	1.712 (0.8224, 3.406)	1.692 (0.8086, 3.513)	1.467 (0.8673, 2.446)	1.355 (0.6038, 2.946)	1.807 (0.8697, 3.667)	1.146 (0.5569, 2.3)	0.8465 (0.4269, 1.626)	1.08 (0.6202, 1.869)	1.085 (0.6259, 1.836)	1.397 (0.6474, 2.925)	**TKI_plus_FUL500**

eTable S3B. PFS effect estimates of ETR patients for pairwise comparisons: hazard ratio (95% credibility interval, CrI) (network 2)

Random effect model

**mTOR_plus_TAM**	1.858 (0.7885, 4.346)	1.751 (0.4054, 7.214)
0.5381 (0.2301, 1.268)	**TAM**	0.9566 (0.3022, 2.927)
0.571 (0.1386, 2.467)	1.045 (0.3417, 3.309)	**TKI_plus_TAM**

Fixed effect model

**mTOR_plus_TAM**	1.849 (1.223, 2.768)	1.759 (0.6957, 4.483)
0.5407 (0.3613, 0.8175)	**TAM**	0.9552 (0.4131, 2.195)
0.5686 (0.2231, 1.437)	1.047 (0.4555, 2.42)	**TKI_plus_TAM**

eTable S3C. OS effect estimates of ETR patients for pairwise comparisons: hazard ratio (95% credibility interval, CrI) (network 1)

**AI**	0.5275 (0.251, 1.129)	1.005 (0.4348, 2.391)	0.7383 (0.4552, 1.189)	0.6681 (0.4669, 0.9571)	1.08 (0.6364, 1.814)	0.8981 (0.5434, 1.499)	1.084 (0.853, 1.373)	1.773 (1.259, 2.503)	0.701 (0.4899, 0.9982)	0.6376 (0.3516, 1.186)	0.784 (0.4648, 1.332)	0.7261 (0.2942, 1.714)
1.896 (0.8861, 3.984)	**AKT_plus_FUL500**	1.919 (0.6054, 5.999)	1.385 (0.7852, 2.453)	1.257 (0.6555, 2.377)	2.028 (0.8051, 5.08)	1.694 (0.9734, 2.926)	2.052 (0.9315, 4.42)	3.351 (1.473, 7.61)	1.328 (0.6052, 2.865)	1.214 (0.617, 2.305)	1.469 (0.8305, 2.588)	1.369 (0.5382, 3.414)
0.9953 (0.4182, 2.3)	0.521 (0.1667, 1.652)	**CDK4_or_6i_plus_AI**	0.7322 (0.2699, 1.934)	0.6583 (0.2601, 1.655)	1.081 (0.3843, 2.885)	0.8903 (0.3278, 2.376)	1.08 (0.4482, 2.578)	1.77 (0.7021, 4.401)	0.6962 (0.2743, 1.731)	0.6315 (0.2249, 1.781)	0.78 (0.2774, 2.082)	0.7265 (0.2004, 2.48)
1.354 (0.8411, 2.197)	0.7223 (0.4077, 1.274)	1.366 (0.5169, 3.706)	**CDK4_or_6i_plus_FUL500**	0.9035 (0.6648, 1.256)	1.465 (0.7036, 2.98)	1.218 (1.045, 1.414)	1.478 (0.866, 2.495)	2.403 (1.324, 4.336)	0.952 (0.5603, 1.635)	0.8677 (0.5969, 1.266)	1.058 (0.8432, 1.332)	0.9929 (0.4735, 1.999)

1.497 (1.045, 2.142)	0.7956 (0.4207, 1.525)	1.519 (0.6041, 3.845)	1.107 (0.7959, 1.504)	**Chemotherapy**	1.623 (0.8432, 3.06)	1.344 (0.938, 1.895)	1.629 (1.058, 2.479)	2.658 (1.614, 4.409)	1.048 (0.694, 1.595)	0.9571 (0.5932, 1.574)	1.174 (0.7976, 1.719)	1.093 (0.4813, 2.377)
0.9259 (0.5511, 1.571)	0.493 (0.1968, 1.242)	0.9251 (0.3466, 2.602)	0.6825 (0.3355, 1.421)	0.616 (0.3268, 1.186)	**FUL250_plus_AI**	0.832 (0.3998, 1.77)	0.9985 (0.5771, 1.795)	1.641 (0.8866, 3.07)	0.6503 (0.3466, 1.204)	0.5881 (0.2666, 1.379)	0.7247 (0.3385, 1.545)	0.6758 (0.2442, 1.823)
1.113 (0.667, 1.84)	0.5903 (0.3418, 1.027)	1.123 (0.4208, 3.051)	0.8209 (0.7071, 0.9571)	0.7439 (0.5277, 1.066)	1.202 (0.565, 2.501)	**FUL500**	1.214 (0.6929, 2.092)	1.981 (1.06, 3.605)	0.7763 (0.4526, 1.363)	0.712 (0.5041, 1.013)	0.8702 (0.7411, 1.031)	0.807 (0.3871, 1.638)
0.9221 (0.7281, 1.172)	0.4873 (0.2262, 1.073)	0.9262 (0.3879, 2.231)	0.6767 (0.4008, 1.155)	0.6141 (0.4034, 0.9447)	1.002 (0.5572, 1.733)	0.8235 (0.478, 1.443)	**MA**	1.627 (1.099, 2.484)	0.6455 (0.4217, 0.9864)	0.5854 (0.314, 1.123)	0.7183 (0.4093, 1.29)	0.6696 (0.2626, 1.645)
0.5641 (0.3994, 0.7941)	0.2984 (0.1314, 0.6787)	0.565 (0.2272, 1.424)	0.4162 (0.2306, 0.7555)	0.3762 (0.2268, 0.6197)	0.6094 (0.3257, 1.128)	0.5049 (0.2774, 0.9436)	0.6146 (0.4025, 0.91)	**MAB_plus_FUL250**	0.395 (0.2437, 0.6491)	0.3609 (0.1828, 0.7412)	0.4394 (0.236, 0.8416)	0.4077 (0.1555, 1.059)
1.426 (1.002, 2.041)	0.7528 (0.3491, 1.652)	1.436 (0.5776, 3.646)	1.05 (0.6117, 1.785)	0.954 (0.627, 1.441)	1.538 (0.8308, 2.885)	1.288 (0.7338, 2.209)	1.549 (1.014, 2.371)	2.532 (1.541, 4.103)	**mTOR**	0.9087 (0.4825, 1.726)	1.119 (0.6295, 1.994)	1.036 (0.4219, 2.589)
1.126 (0.9292, 1.386)	0.5943 (0.2927, 1.238)	1.133 (0.4788, 2.792)	0.8275 (0.5387, 1.281)	0.7517 (0.5595, 1.014)	1.214 (0.6958, 2.119)	1.011 (0.6362, 1.605)	1.22 (0.8971, 1.665)	1.999 (1.365, 2.963)	0.7867 (0.5867, 1.049)	0.7176 (0.4094, 1.279)	0.8808 (0.5439, 1.437)	0.8214 (0.3413, 1.885)
1.568 (0.8429, 2.845)	0.8239 (0.4339, 1.621)	1.583 (0.5615, 4.447)	1.152 (0.79, 1.675)	1.045 (0.6352, 1.686)	1.7 (0.7254, 3.751)	1.404 (0.9874, 1.984)	1.708 (0.8902, 3.185)	2.771 (1.349, 5.471)	1.1 (0.5794, 2.073)	**mTOR_plus_FUL500**	1.218 (0.8281, 1.799)	1.141 (0.4925, 2.507)
1.276 (0.751, 2.152)	0.6806 (0.3863, 1.204)	1.282 (0.4804, 3.604)	0.9451 (0.7509, 1.186)	0.8521 (0.5817, 1.254)	1.38 (0.6474, 2.954)	1.149 (0.9704, 1.349)	1.392 (0.7753, 2.443)	2.276 (1.188, 4.237)	0.8939 (0.5014, 1.589)	0.8208 (0.556, 1.208)	**PI3Ki_plus_FUL500**	0.9359 (0.4362, 1.912)
1.377 (0.5835, 3.399)	0.7304 (0.293, 1.858)	1.376 (0.4032, 4.99)	1.007 (0.5004, 2.112)	0.9148 (0.4207, 2.078)	1.48 (0.5486, 4.095)	1.239 (0.6105, 2.583)	1.493 (0.6079, 3.807)	2.453 (0.9445, 6.431)	0.965 (0.3862, 2.37)	0.8768 (0.3989, 2.03)	1.068 (0.523, 2.292)	**TKI_plus_FUL500**

eTable S3D. OS effect estimates of ETR patients for pairwise comparisons: hazard ratio (95% credibility interval, CrI) (network 2)

**mTOR_plus_TAM**	2.214 (0.7181, 6.633)
0.4518 (0.1508, 1.393)	**TAM**

eTable S4. SUCRA values of PFS for CDK4/6 inhibitors in ETS patients

Treatment	Sucra value FE
Abe_plus_AI	0.9430
Pal_plus_AI	0.7157
Rib_plus_AI	0.6805
Pal_plus_Ful	0.5872
Rib_plus_Ful	0.3573
AI	0.2139
Ful	0.0023

eTable S5. Effect estimates of PFS for CDK4/6 inhibitors in ETS patients：hazard ratio (95% credibility interval, CrI)

**Abe_plus_AI**	1.905 (1.522, 2.392)	2.991 (1.886, 4.688)	1.22 (0.9062, 1.658)	1.35 (0.9114, 1.96)	1.245 (0.842, 1.852)	1.657 (0.9602, 2.776)
0.5248 (0.418, 0.6568)	**AI**	1.572 (1.046, 2.329)	0.6409 (0.5275, 0.7793)	0.7064 (0.51, 0.9607)	0.6541 (0.4729, 0.9063)	0.8683 (0.529, 1.388)
0.3343 (0.2133, 0.5303)	0.6361 (0.4293, 0.9557)	**Ful**	0.4096 (0.2943, 0.5851)	0.4506 (0.3554, 0.5682)	0.4151 (0.2541, 0.7098)	0.5511 (0.4189, 0.7195)
0.8195 (0.603, 1.104)	1.56 (1.283, 1.896)	2.442 (1.709, 3.398)	**Pal_plus_AI**	1.1 (0.85, 1.407)	1.018 (0.6985, 1.492)	1.351 (0.8656, 2.074)
0.7408 (0.5103, 1.097)	1.416 (1.041, 1.961)	2.219 (1.76, 2.814)	0.9091 (0.7109, 1.177)	**Pal_plus_Ful**	0.9306 (0.5946, 1.481)	1.226 (0.8575, 1.755)
0.8035 (0.5401, 1.188)	1.529 (1.103, 2.115)	2.409 (1.409, 3.936)	0.9824 (0.6701, 1.432)	1.075 (0.6752, 1.682)	**Rib_plus_AI**	1.319 (0.7262, 2.369)
0.6035 (0.3602, 1.041)	1.152 (0.7206, 1.89)	1.815 (1.39, 2.387)	0.7401 (0.4823, 1.155)	0.8154 (0.5699, 1.166)	0.7581 (0.4221, 1.377)	**Rib_plus_Ful**

eTable S6. SUCRA values for PI3ki in ETS patients

Treatment	Sucra value FE
Bup_plus_Ful	0.7972
Alp_plus_Ful	0.5011
Ful	0.2017

eTable S7. Effect estimates of PFS for PI3ki in ETS patients hazard ratio (95% credibility interval, CrI)

**Alp_plus_Ful**	0.8673 (0.2995, 2.474)	1.156 (0.4371, 3)
1.153 (0.4042, 3.338)	**Bup_plus_Ful**	1.334 (0.8531, 2.074)
0.865 (0.3333, 2.288)	0.7496 (0.4822, 1.172)	**Ful**

eTable S8. SUCRA values of PFS for HDACi in ETS patients

Treatment	Sucra value FE
Tuc_plus_AI	0.8886
Ent_plus_AI	0.4870
AI	0.1244

eTable S9. Effect estimates of PFS for HDACi in ETS patients hazard ratio (95% credibility interval, CrI)

**AI**	0.8521 (0.4475, 1.617)	0.6817 (0.3877, 1.191)
1.174 (0.6185, 2.235)	**Ent_plus_AI**	0.8012 (0.3419, 1.878)
1.467 (0.84, 2.58)	1.248 (0.5326, 2.925)	**Tuc_plus_AI**

eTable S10. SUCRA of PFS for visceral metastases in ETS patients

Treatment	Sucra value FE
CDK4_or_6i_plus_AI	0.9172
CDK4_or_6i_plus_Ful_500	0.7795

Tam	0.6726
AI_plus_MAB	0.6181
Ful_500	0.2008
AI	0.1881
Ful	0.1237

eTable S11. Effect estimates of PFS for visceral metastases in ETS patients：hazard ratio (95% credibility interval, CrI)

**AI**	0.6908 (0.5272, 0.904)	0.5562 (0.4414, 0.7032)	0.6018 (0.4576, 0.7973)	1.051 (0.8186, 1.355)	0.9887 (0.7402, 1.325)	0.6575 (0.5399, 0.7982)
1.448 (1.106, 1.897)	**AI_plus_MAB**	0.8052 (0.5635, 1.148)	0.8723 (0.5919, 1.283)	1.524 (1.055, 2.188)	1.432 (0.9619, 2.131)	0.9513 (0.6843, 1.327)
1.798 (1.422, 2.266)	1.242 (0.8708, 1.775)	**CDK4_or_6i_plus_AI**	1.083 (0.8681, 1.351)	1.89 (1.424, 2.516)	1.778 (1.224, 2.58)	1.181 (0.8693, 1.596)
1.662 (1.254, 2.186)	1.146 (0.7793, 1.689)	0.9234 (0.7404, 1.152)	**CDK4_or_6i_plus_Ful_500**	1.748 (1.333, 2.283)	1.643 (1.096, 2.459)	1.092 (0.776, 1.526)
0.9513 (0.7378, 1.222)	0.6562 (0.4571, 0.9478)	0.5292 (0.3974, 0.7025)	0.572 (0.438, 0.7501)	**Ful**	0.9411 (0.6408, 1.383)	0.6253 (0.4534, 0.8568)
1.011 (0.7546, 1.351)	0.6981 (0.4693, 1.04)	0.5624 (0.3876, 0.8172)	0.6086 (0.4067, 0.9123)	1.063 (0.7231, 1.561)	**Ful_500**	0.6645 (0.4669, 0.9431)
1.521 (1.253, 1.852)	1.051 (0.7534, 1.461)	0.8464 (0.6264, 1.15)	0.9161 (0.6552, 1.289)	1.599 (1.167, 2.205)	1.505 (1.06, 2.142)	**Tam**

eTable S12. SUCRA values of PFS for bone metastases in ETS patients

Treatment	Sucra value FE
CDK4_or_6i_plus_Ful_500	0.9019
CDK4_or_6i_plus_AI	0.8726
Ful	0.4496
Tam	0.4397
AI_plus_MAB	0.1995
AI	0.1367

eTable S13. Effect estimates of PFS for bone metastases in ETS patients：hazard ratio (95% credibility interval, CrI)

**AI**	0.9878 (0.5946, 1.646)	0.4418 (0.2824, 0.6906)	0.4284 (0.2391, 0.7655)	0.7174 (0.3415, 1.494)	0.7937 (0.6431, 0.9828)
1.012 (0.6074, 1.682)	**AI_plus_MAB**	0.4477 (0.2244, 0.878)	0.4345 (0.2011, 0.9366)	0.7248 (0.2959, 1.771)	0.8051 (0.4635, 1.394)
2.264 (1.448, 3.541)	2.234 (1.139, 4.456)	**CDK4_or_6i_plus_AI**	0.9721 (0.673, 1.406)	1.623 (0.9076, 2.895)	1.798 (1.1, 2.962)
2.334 (1.306, 4.183)	2.301 (1.068, 4.974)	1.029 (0.7113, 1.486)	**CDK4_or_6i_plus_Ful_500**	1.67 (1.062, 2.626)	1.848 (0.9934, 3.449)
1.394 (0.6693, 2.929)	1.38 (0.5648, 3.38)	0.616 (0.3454, 1.102)	0.5989 (0.3808, 0.9419)	**Ful**	1.107 (0.514, 2.397)
1.26 (1.018, 1.555)	1.242 (0.7172, 2.158)	0.5563 (0.3376, 0.9091)	0.541 (0.29, 1.007)	0.9032 (0.4171, 1.946)	**Tam**

eTable S14. SUCRA values of PFS for CDK4/6 inhibitors in ETR patients (Network 1)

Treatment	Sucra value FE
Dal_plus_FUL500	0.8995
Abe_plus_FUL500	0.6545
Chemotherapy	0.5685
Rib_plus_FUL500	0.5032
Pal_plus_FUL500	0.3587
FUL500	0.015

eTable S15. Effect estimates of PFS for CDK4/6 inhibitors in ETR patients：hazard ratio (95% credibility interval, CrI) (Network 1)

**Abe_plus_FUL500**	1.094 (0.604, 2.011)	0.8141 (0.5675, 1.169)	1.937 (1.597, 2.327)	1.238 (0.7264, 2.126)	1.108 (0.7986, 1.516)
0.9142 (0.4973, 1.656)	**Chemotherapy**	0.7458 (0.387, 1.409)	1.764 (0.9818, 3.16)	1.128 (0.8451, 1.498)	1.013 (0.5314, 1.907)
1.228 (0.8558, 1.762)	1.341 (0.7099, 2.584)	**Dal_plus_FUL500**	2.386 (1.749, 3.217)	1.514 (0.8438, 2.757)	1.359 (0.9176, 1.997)
0.5162 (0.4297, 0.626)	0.5668 (0.3164, 1.019)	0.4191 (0.3108, 0.5719)	**FUL500**	0.636 (0.3837, 1.066)	0.5719 (0.4389, 0.7403)
0.808 (0.4703, 1.377)	0.8867 (0.6677, 1.183)	0.6603 (0.3628, 1.185)	1.572 (0.9378, 2.606)	**Pal_plus_FUL500**	0.8949 (0.5068, 1.589)
0.9023 (0.6595, 1.252)	0.9869 (0.5243, 1.882)	0.7357 (0.5006, 1.09)	1.749 (1.351, 2.278)	1.117 (0.6292, 1.973)	**Rib_plus_FUL500**

eTable S16. SUCRA values of PFS for CDK4/6 inhibitors in ETR patients (Network 2)

Treatment	Sucra value FE
Rib_plus_AI	0.9266
Pal_plus_AI	0.5732

AI	0.0001

eTable S17. Effect estimates of PFS for CDK4/6 inhibitors in ETR patients：hazard ratio (95% credibility interval, CrI) (Network 2)

**AI**	0.5165 (0.3513, 0.7492)	0.377 (0.2357, 0.5942)
1.936 (1.335, 2.846)	**Pal_plus_AI**	0.7318 (0.4067, 1.311)
2.653 (1.683, 4.243)	1.367 (0.7628, 2.459)	**Rib_plus_AI**

eTable S18. SUCRA values of PFS for PI3ki in ETR patients

Treatment	Sucra value FE
Bup_plus_FUL500	0.7573
Alp_plus_FUL500	0.7078
Pic_plus_FUL500	0.4702
FUL500	0.0647

eTable S19. Effect estimates of PFS for PI3ki in ETR patients：hazard ratio (95% credibility interval, CrI)

**Alp_plus_FUL500**	1.054 (0.504, 2.253)	1.573 (0.7549, 3.328)	1.248 (0.5724, 2.751)
0.9489 (0.4438, 1.984)	**Bup_plus_FUL500**	1.496 (1.187, 1.858)	1.196 (0.8046, 1.759)
0.6358 (0.3004, 1.325)	0.6686 (0.5383, 0.8427)	**FUL500**	0.8002 (0.58, 1.092)
0.8014 (0.3635, 1.747)	0.8361 (0.5684, 1.243)	1.25 (0.9154, 1.724)	**Pic_plus_FUL500**

eTable S20. SUCRA values of PFS for HDACi in ETR patients

Treatment	Sucra value FE
Ent_plus_AI	0.9590
Tuc_plus_AI	0.3507
AI	0.1903

eTable S21. Effect estimates of PFS for HDACi in ETR patients：hazard ratio (95% credibility interval, CrI)

**AI**	0.4713 (0.1493, 1.486)	0.9185 (0.3334, 2.554)
2.122 (0.6729, 6.699)	**Ent_plus_AI**	1.944 (0.4203, 9.124)
1.089 (0.3915, 3)	0.5143 (0.1096, 2.379)	**Tuc_plus_AI**

eTable S22. SUCRA values of PFS for visceral metastases in ETR patients (network 1)

Treatment	Sucra value FE
CDK4_OR_6i_plus_FUL500	0.9999
PI3KI_plus_Ful500	0.4998
FUL500	0.0002

eTable S23. Effect estimates of PFS for visceral metastases in ETR patients：hazard ratio (95% credibility interval, CrI) (network 1)

**CDK4_OR_6i_plus_FUL500**	2.12 (1.733, 2.597)	1.607 (1.239, 2.091)
0.4717 (0.3851, 0.5772)	**FUL500**	0.7582 (0.6406, 0.8958)
0.6221 (0.4782, 0.8071)	1.319 (1.116, 1.561)	**PI3KI_plus_Ful500**

eTable S24. SUCRA values of PFS for visceral metastases in ETR patients (network 2)

Treatment	Sucra value using RE
mTOR_plus_AI	0.9497
AI	0.0502

eTable S25. Effect estimates of PFS for visceral metastases in ETR patients：hazard ratio (95% credibility interval, CrI) (network 2)

**AI**	0.4702 (0.1801, 1.244)
2.127 (0.8041, 5.553)	**mTOR_plus_AI**

eTable S26. SUCRA values of PFS for bone metastases in ETR patients (network 1)

Treatment	Sucra value FE
CDK4_OR_6i_plus_FUL500	0.9364
PI3K_plus_Ful500	0.5602
FUL500	0.0033

eTable S27. Effect estimates of PFS for bone metastases in ETR patients：hazard ratio (95% credibility interval, CrI) (network 1)

**CDK4_OR_6i_plus_FUL500**	1.932 (1.379, 2.71)	1.307 (0.8307, 2.057)
0.5177 (0.369, 0.7253)	**FUL500**	0.6766 (0.4989, 0.9202)
0.7649 (0.4862, 1.204)	1.478 (1.087, 2.004)	**PI3K_plus_Ful500**

eTable S28. SUCRA values of PFS for bone metastases in ETR patients (network 2)

Treatment	Sucra value RE
mTOR_plus_AI	0.9469
AI	0.053

eTable S29. Effect estimates of PFS for bone metastases in ETR patients：hazard ratio (95% credibility interval, CrI) (network 2)

**AI**	0.3285 (0.07777, 1.411)
3.044 (0.7089, 12.86)	**mTOR_plus_AI**

eTable S30. SUCRA values of PFS for post-menopausal women in ETR patients (network 1)

Treatment	Sucra value FE
Chemotherapy	0.9633
CDK4_OR_6i_plus_FUL500	0.8865
mTOR_plus_AI	0.8111
AKT_plus_FUL	0.7800
CDK4_OR_6i_plus_AI	0.7329
TKI_plus_FUL500	0.6446
mTOR_plus_FUL500	0.5891
PI3Ki_plus_Ful500	0.5846
mTOR	0.5672
HDAC_plus_AI	0.4169
TKI_plus_AI	0.3991
FUL500	0.3036
FUL_250_plus_AI	0.2010
FUL250	0.2000
Anti_androgen_plus_AI	0.1518
MA	0.1368
AI	0.1309

eTable S31. Effect estimates of PFS for post-menopausal women in ETR patients：hazard ratio (95% credibility interval, CrI) (network 1)

**AI**	0.475 (0.2672, 0.8634)	1.019 (0.6635, 1.581)	0.5106 (0.3529, 0.7428)	0.4177 (0.2755, 0.6317)	0.3694 (0.2676, 0.502)	0.9463 (0.7917, 1.138)	0.9496 (0.7298, 1.241)	0.823 (0.5323, 1.261)	0.7628 (0.5176, 1.136)	0.6304 (0.4596, 0.8501)	0.4665 (0.3993, 0.5453)	0.6027 (0.3732, 0.9782)	0.6081 (0.3823, 0.9538)	0.7801 (0.6234, 0.9682)	0.558 (0.2813, 1.094)
2.105 (1.158, 3.742)	**AKT_plus_FUL**	2.141 (1.046, 4.366)	1.076 (0.5481, 2.145)	0.8779 (0.5859, 1.31)	0.7739 (0.47, 1.276)	1.999 (1.086, 3.667)	2.006 (1.037, 3.771)	1.722 (1.181, 2.506)	1.611 (0.8076, 3.187)	1.322 (0.7012, 2.48)	0.9805 (0.5562, 1.73)	1.269 (0.8298, 1.959)	1.275 (0.8683, 1.887)	1.643 (0.8618, 3.055)	1.179 (0.6226, 2.187)
0.981 (0.6327, 1.507)	0.4671 (0.229, 0.9557)	**Anti_androgen_plus_AI**	0.5006 (0.2883, 0.8801)	0.4097 (0.222, 0.7492)	0.3626 (0.2093, 0.6072)	0.9292 (0.5769, 1.48)	0.9286 (0.5567, 1.527)	0.8027 (0.4374, 1.486)	0.7492 (0.4163, 1.326)	0.6139 (0.3619, 1.044)	0.4576 (0.2879, 0.7194)	0.5947 (0.3165, 1.133)	0.596 (0.3197, 1.122)	0.7669 (0.4757, 1.22)	0.5538 (0.2399, 1.243)

1.958 (1.346, 2.834)	0.9297 (0.4663, 1.825)	1.998 (1.136, 3.468)	**CDK4_or_6i_plus_AI**	0.8174 (0.4718, 1.41)	0.7244 (0.4481, 1.161)	1.852 (1.224, 2.813)	1.853 (1.184, 2.934)	1.604 (0.9108, 2.835)	1.494 (0.8582, 2.552)	1.228 (0.7536, 1.994)	0.9116 (0.6093, 1.354)	1.179 (0.6584, 2.167)	1.188 (0.6628, 2.132)	1.515 (0.9755, 2.363)	1.091 (0.507, 2.38)
2.394 (1.583, 3.63)	1.139 (0.7636, 1.707)	2.441 (1.335, 4.505)	1.223 (0.709, 2.119)	**CDK4_or_6i_plus_FUL500**	0.886 (0.6608, 1.176)	2.273 (1.457, 3.56)	2.283 (1.385, 3.723)	1.969 (1.696, 2.28)	1.827 (1.057, 3.171)	1.499 (0.9374, 2.417)	1.116 (0.7568, 1.658)	1.449 (1.143, 1.847)	1.454 (1.217, 1.748)	1.861 (1.17, 3.007)	1.33 (0.7786, 2.317)
2.707 (1.992, 3.737)	1.292 (0.7837, 2.128)	2.758 (1.647, 4.777)	1.38 (0.8612, 2.232)	1.129 (0.8504, 1.513)	**Chemotherapy**	2.578 (1.789, 3.669)	2.57 (1.719, 3.854)	2.228 (1.611, 3.037)	2.058 (1.272, 3.39)	1.697 (1.158, 2.496)	1.264 (0.9635, 1.668)	1.645 (1.125, 2.367)	1.642 (1.178, 2.284)	2.1 (1.439, 3.12)	1.507 (0.819, 2.788)
1.057 (0.8789, 1.263)	0.5002 (0.2727, 0.9205)	1.076 (0.6757, 1.733)	0.54 (0.3555, 0.817)	0.4399 (0.2809, 0.6863)	0.3878 (0.2725, 0.559)	**FUL250**	1 (0.8262, 1.211)	0.8641 (0.5393, 1.388)	0.8058 (0.5278, 1.243)	0.6635 (0.4622, 0.9489)	0.4925 (0.3873, 0.6274)	0.6371 (0.3805, 1.06)	0.6401 (0.3904, 1.031)	0.8231 (0.6133, 1.097)	0.5879 (0.2938, 1.182)
1.053 (0.8056, 1.37)	0.4985 (0.2652, 0.9642)	1.077 (0.655, 1.796)	0.5396 (0.3409, 0.8444)	0.4381 (0.2686, 0.7218)	0.389 (0.2595, 0.5817)	0.9997 (0.8259, 1.21)	**FUL250_plus_AI**	0.8632 (0.5191, 1.432)	0.8065 (0.4993, 1.28)	0.6661 (0.4409, 0.9857)	0.4917 (0.3624, 0.6622)	0.6337 (0.3676, 1.104)	0.6378 (0.3777, 1.074)	0.8227 (0.5808, 1.161)	0.5839 (0.2821, 1.242)
1.215 (0.793, 1.879)	0.5807 (0.3991, 0.8471)	1.246 (0.6732, 2.286)	0.6235 (0.3527, 1.098)	0.5078 (0.4387, 0.5895)	0.4489 (0.3293, 0.6207)	1.157 (0.7206, 1.854)	1.158 (0.6982, 1.926)	**FUL500**	0.9274 (0.5265, 1.64)	0.7667 (0.4607, 1.261)	0.5669 (0.3742, 0.8561)	0.7351 (0.6089, 0.8863)	0.7401 (0.668, 0.8185)	0.9462 (0.5863, 1.553)	0.6753 (0.4019, 1.146)
1.311 (0.8802, 1.932)	0.6206 (0.3138, 1.238)	1.335 (0.7541, 2.402)	0.6693 (0.3919, 1.165)	0.5472 (0.3154, 0.9457)	0.486 (0.295, 0.7864)	1.241 (0.8045, 1.895)	1.24 (0.7812, 2.003)	1.078 (0.6099, 1.899)	**HDAC_plus_AI**	0.8261 (0.501, 1.35)	0.6121 (0.3994, 0.9222)	0.7925 (0.4338, 1.457)	0.7961 (0.4443, 1.406)	1.021 (0.6489, 1.58)	0.7339 (0.3345, 1.596)
0.9897 (0.7945, 1.24)	0.4679 (0.2536, 0.8787)	1.007 (0.6295, 1.629)	0.5054 (0.3313, 0.7814)	0.413 (0.2603, 0.6556)	0.3653 (0.2502, 0.5321)	0.936 (0.7105, 1.243)	0.9387 (0.6636, 1.325)	0.8093 (0.4981, 1.305)	0.7598 (0.478, 1.186)	0.6224 (0.4291, 0.9031)	0.4625 (0.3543, 0.6013)	0.594 (0.3494, 1.01)	0.6012 (0.3634, 0.9882)	0.769 (0.5668, 1.066)	0.5494 (0.2704, 1.132)
1.586 (1.176, 2.176)	0.7562 (0.4032, 1.426)	1.629 (0.9576, 2.763)	0.8146 (0.5015, 1.327)	0.6673 (0.4138, 1.067)	0.5892 (0.4006, 0.8638)	1.507 (1.054, 2.164)	1.501 (1.014, 2.268)	1.304 (0.7931, 2.171)	1.211 (0.7406, 1.996)	**mTOR**	0.7426 (0.5689, 0.9624)	0.96 (0.5722, 1.629)	0.9691 (0.5844, 1.611)	1.237 (0.847, 1.824)	0.8814 (0.4316, 1.814)
2.144 (1.834, 2.504)	1.02 (0.5779, 1.798)	2.185 (1.39, 3.473)	1.097 (0.7386, 1.641)	0.8959 (0.6033, 1.321)	0.791 (0.5996, 1.038)	2.03 (1.594, 2.582)	2.034 (1.51, 2.759)	1.764 (1.168, 2.672)	1.634 (1.084, 2.504)	1.347 (1.039, 1.758)	**mTOR_plus_AI**	1.3 (0.818, 2.063)	1.304 (0.8539, 2.005)	1.673 (1.281, 2.195)	1.193 (0.6117, 2.328)

1.659 (1.022, 2.68)	0.788 (0.5105, 1.205)	1.681 (0.8827, 3.159)	0.848 (0.4615, 1.519)	0.6902 (0.5415, 0.8751)	0.6079 (0.4225, 0.889)	1.57 (0.943, 2.628)	1.578 (0.906, 2.72)	1.36 (1.128, 1.642)	1.262 (0.6862, 2.305)	1.042 (0.6137, 1.748)	0.7691 (0.4847, 1.222)	**mTOR_plus_FUL500**	1.004 (0.8112, 1.251)	1.284 (0.7586, 2.192)	0.9183 (0.5351, 1.599)
1.644 (1.048, 2.616)	0.7842 (0.5301, 1.152)	1.678 (0.8914, 3.128)	0.8419 (0.4691, 1.509)	0.6878 (0.5722, 0.8215)	0.6089 (0.4378, 0.8489)	1.562 (0.9697, 2.562)	1.568 (0.9309, 2.648)	1.351 (1.222, 1.497)	1.256 (0.7112, 2.251)	1.032 (0.6208, 1.711)	0.7666 (0.4988, 1.171)	0.9961 (0.7996, 1.233)	**PI3Ki_plus_FUL500**	1.282 (0.7792, 2.117)	0.9136 (0.5422, 1.534)
1.282 (1.033, 1.604)	0.6085 (0.3274, 1.16)	1.304 (0.8195, 2.102)	0.6601 (0.4232, 1.025)	0.5374 (0.3325, 0.8547)	0.4761 (0.3205, 0.6949)	1.215 (0.9115, 1.63)	1.216 (0.861, 1.722)	1.057 (0.644, 1.706)	0.9798 (0.6327, 1.541)	0.8082 (0.5482, 1.181)	0.5979 (0.4557, 0.7806)	0.7791 (0.4563, 1.318)	0.7803 (0.4724, 1.283)	**TKI_plus_AI**	0.7165 (0.348, 1.473)
1.792 (0.9145, 3.555)	0.848 (0.4573, 1.606)	1.806 (0.8047, 4.168)	0.9165 (0.4202, 1.972)	0.7516 (0.4316, 1.284)	0.6635 (0.3586, 1.221)	1.701 (0.8461, 3.404)	1.713 (0.8051, 3.545)	1.481 (0.8729, 2.488)	1.363 (0.6265, 2.99)	1.135 (0.5513, 2.317)	0.8383 (0.4295, 1.635)	1.089 (0.6252, 1.869)	1.095 (0.6517, 1.844)	1.396 (0.6789, 2.874)	**TKI_plus_FUL500**

eTable S32. SUCRA values of PFS for post-menopausal women in ETR patients (network 2)

Treatment	Sucra value FE
mTOR_plus_TAM	0.9912
TAM	0.4101
TKI_plus_TAM	0.0986

eTable S33. Effect estimates of PFS for post-menopausal women in ETR patients：hazard ratio (95% credibility interval, CrI) (network 2)

**mTOR_plus_TAM**	1.848 (1.237, 2.738)	2.66 (0.1928, 35.56)
0.5411 (0.3652, 0.8086)	**TAM**	1.479 (0.1013, 18.21)
0.3759 (0.02812, 5.188)	0.6761 (0.05491, 9.876)	**TKI_plus_TAM**

eTable S34. SUCRA values of PFS for pre-menopausal women in ETR patients (network 1)

Treatment	Sucra value RE
CDK4_OR_6_plus_Ful500	0.955

Ful_500	0.045

eTable S35. Effect estimates of PFS for pre-menopausal women in ETR patients：hazard ratio (95% credibility interval, CrI) (network 1)

**CDK4_or_6i_plus_FUL500**	1.867 (0.8317, 4.299)
0.5355 (0.2326, 1.202)	**FUL500**

eTable S36. SUCRA values of PFS for pre-menopausal women in ETR patients (network 2)

Treatment	Sucra value RE
mTOR_plus_AI	0.9427
AI	0.0572

eTable S37. Effect estimates of PFS for pre-menopausal women in ETR patients：hazard ratio (95% credibility interval, CrI) (network 2)

**AI**	0.5281 (0.2249, 1.257)
1.894 (0.7958, 4.446)	**mTOR_plus_AI**

eFigure S1. Risk of bias across studies (main analysis in ETS and ETR patients)

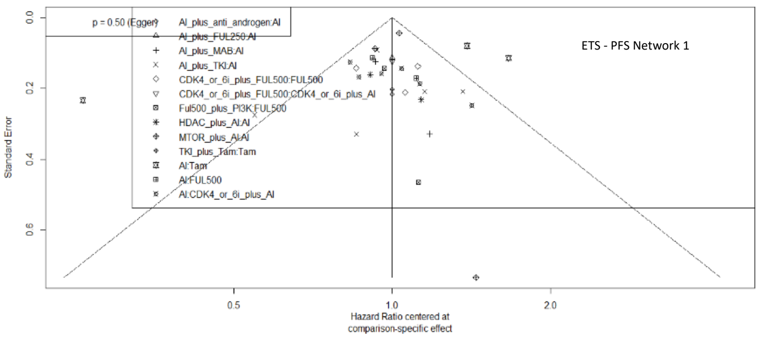
ETS - PFS Network 1
p = 0.50 (Egger)
AI_plus_anti_androgen:AI
AI_plus_FUL250:AI
AI_plus_MAB:AI
AI_plus_TKI:AI
CDK4_or_6i_plus_FUL500:FUL500
CDK4_or_6i_plus_FUL500:CDK4_or_6i_plus_AI
Ful500_plus_PI3K:FUL500
HDAC_plus_AI:AI
MTOR_plus_AI:AI
TKI_plus_Tam:Tam
AI:Tam
AI:FUL500
AI:CDK4_or_6i_plus_AI
Standard Error
Hazard Ratio centered at comparison-specific effect

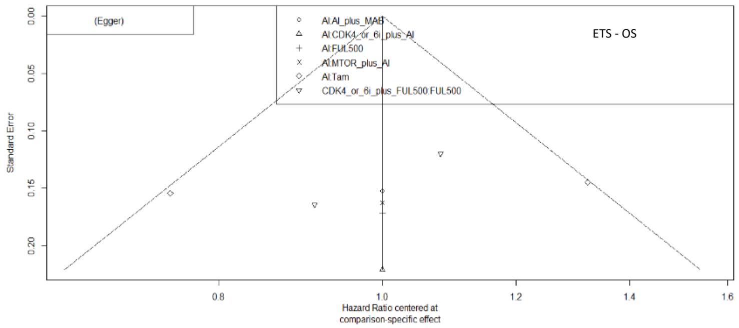
ETS - OS
(Egger)
AI:AI_plus_MAB
AI:CDK4_or_6i_plus_AI
AI:FUL500
AI:MTOR_plus_AI
AI:Tam
CDK4_or_6i_plus_FUL500:FUL500
Standard Error
Hazard Ratio centered at comparison-specific effect

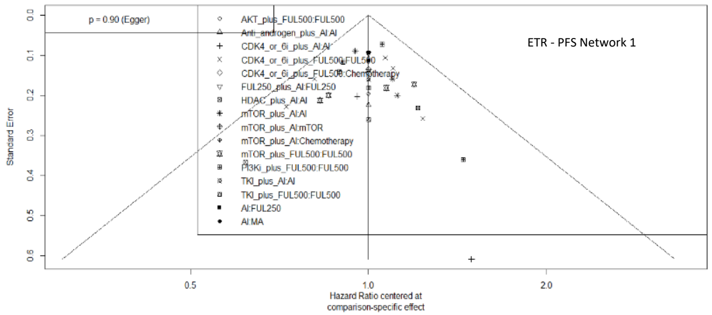
ETR - PFS Network 1
p = 0.90 (Egger)
AKT_plus_FUL500:FUL500
Anti_androgen_plus_AI:AI
CDK4_or_6i_plus_AI:AI
CDK4_or_6i_plus_FUL500:FUL500
CDK4_or_6i_plus_FUL500:Chemotherapy
FUL250_plus_AI:FUL250
HDAC_plus_AI:AI
mTOR_plus_AI:AI
mTOR_plus_AI:mTOR
mTOR_plus_AI:Chemotherapy
mTOR_plus_FUL500:FUL500
PI3Ki_plus_FUL500:FUL500
TKI_plus_AI:AI
TKI_plus_FUL500:FUL500
AI:FUL250
AI:MA
Standard Error
Hazard Ratio centered at comparison-specific effect

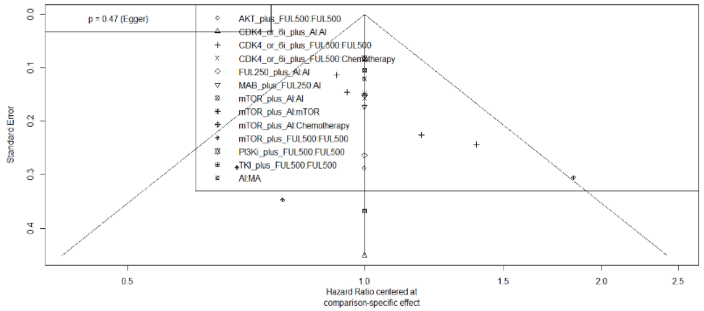
p = 0.47 (Egger)
AKT_plus_FUL500:FUL500
CDK4_or_6i_plus_AI:AI
CDK4_or_6i_plus_FUL500:FUL500
CDK4_or_6i_plus_FUL500:Chemotherapy
FUL250_plus_AI:AI
MAB_plus_FUL250:AI
mTOR_plus_AI:AI
mTOR_plus_AI:mTOR
mTOR_plus_AI:Chemotherapy
mTOR_plus_FUL500:FUL500
PI3Ki_plus_FUL500:FUL500
TKI_plus_FUL500:FUL500
AI:MA
Standard Error
Hazard Ratio centered at comparison-specific effect

eFigure S2. Risk of bias across studies (subgroup analysis for targeted regimens in ETS patients)

ETS CDK4/6I

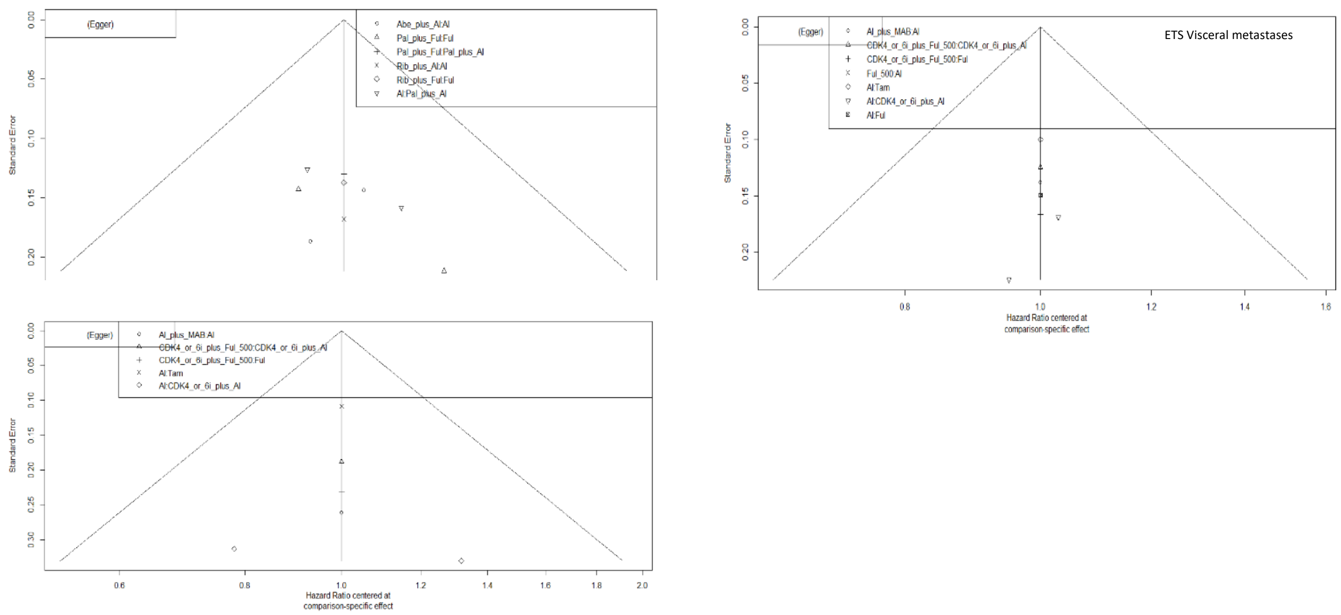

S Bone metastases

eFigure S3. Risk of bias across studies (subgroup analysis for targeted regimens and menopausal women in ETR patients)

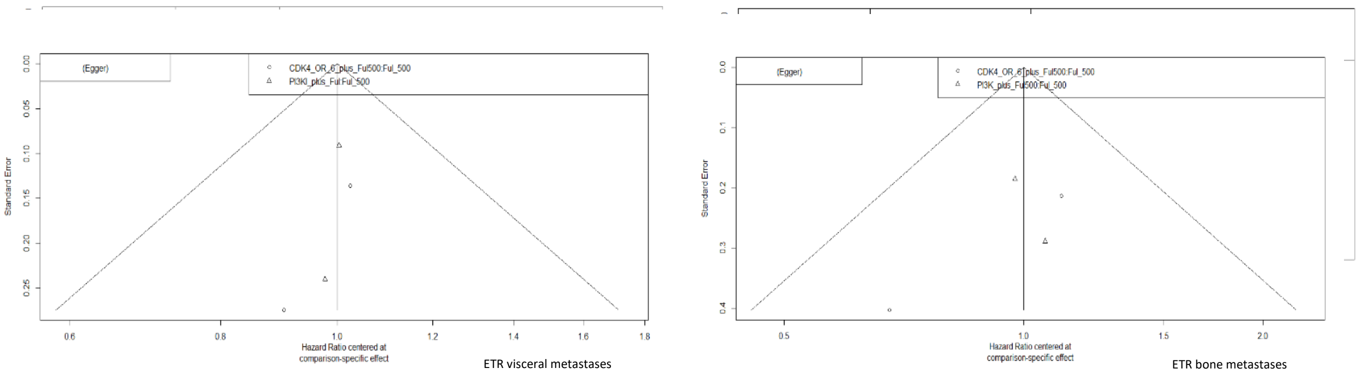

ETR visceral metastases

ETR bone metastases

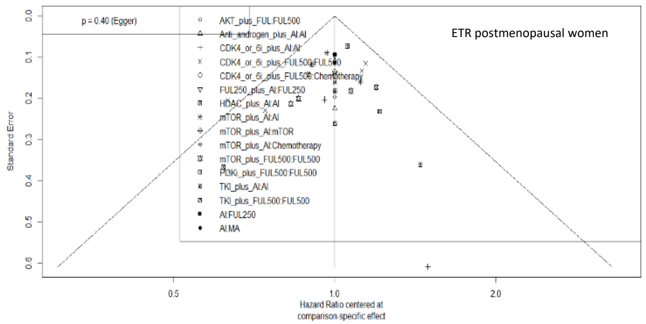

eFigure S4. Quality assessment data for individual studies

Study name	D1	D2	D3	D4	D5	Overall
Ibrahim 2011	+	+	+	+	!	!
Goetz 2017	+	+	+	+	+	+
Zhang 2020	+	+	+	+	+	+
Antonio 2013	+	+	!	+	!	!
Dickler 2016	+	-	+	-	!	-
Goss 2007	+	+	+	+	+	+
Hyams	!	!	+	!	+	-
Cristofanilli 2010	+	+	+	+	+	+
Johnston 2009	+	+	+	+	+	+
Johnston 2016	+	+	+	+	+	+
Krop 2020	+	+	+	+	+	+
Albanell 2020	+	+	+	!	+	!
Llombart-Cussac 2021	!	!	+	-	+	-
Mauridsen	-	!	!	-	-	-
Paridaens 2008	+	!	+	!	+	!
Milla Santos 2003	+	!	+	+	+	!
Paul 2019	!	!	+	!	+	!
Mehta 2012	+	+	+	+	+	+
Finn 2015	+	+	+	+	+	+
Finn 2016	-	!	+	+	+	-
Cristofanilli 2016	+	+	+	+	+	+
Hortobagyi 3028	+	+	+	+	+	+
Slamon 2021	+	+	+	+	+	+
Johnston 2013	+	+	+	+	+	+
Andre 2019	+	+	+	+	+	+
Leo 2018	+	+	+	+	+	+
Fan 2021	+	+	+	+	+	+
Osborne 2011	+	+	+	+	+	+
Jiang 2019	+	+	+	+	+	+
Yardley 2011	+	+	+	+	+	+
Xu 2021	+	+	+	+	+	+
Treilleux 2015	+	+	+	+	+	+
Baselga 2017	+	+	+	+	+	+
Piccart 2014	+	+	+	+	+	+
Jones 2020	+	+	+	+	+	+
Krop 2016	+	+	+	+	+	+
Jerusalem 2018	!	!	+	-	-	-
Schmid 2019	+	+	+	+	+	+
Mssolino 2017	+	+	+	+	+	+
Martin 2021	-	-	+	-	+	-
Kornblum 2018	+	+	+	+	+	+
Xu 2021	+	+	+	+	+	+
Buzdar 2002	+	+	+	+	+	+
Sledge 2017	+	+	+	+	+	+
Shao 2021	+	+	+	+	+	+
Robertson 2012	!	+	+	+	!	!
Robertson 2016	+	+	+	+	!	+

+	Low risk	D1	Randomisation process
!	Some concerns	D2	Deviations from the intended i
-	High risk	D3	Missing outcome data
		D4	Measurement of the outcome
		D5	Selection of the reported resul

eFigure S5. Chemical structure of fulvestrant

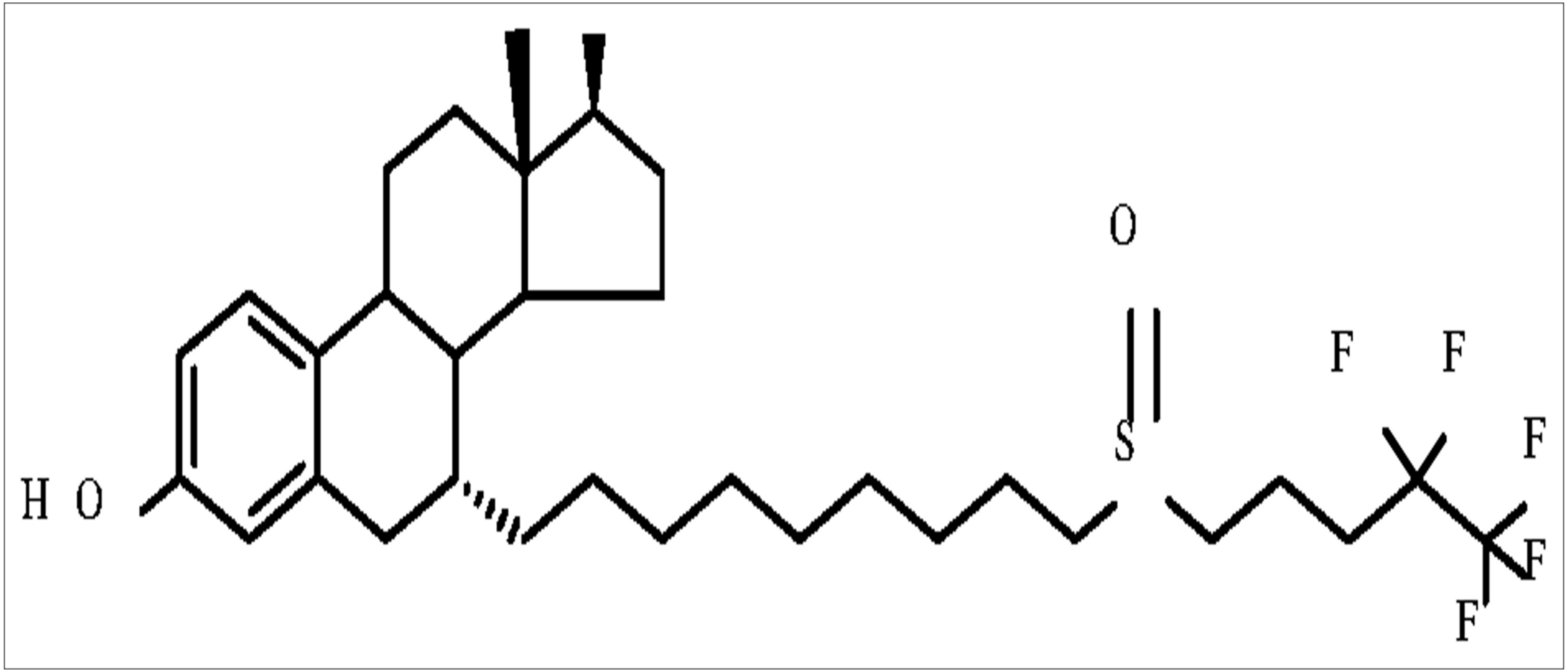
HO
O
S
F
F
F
F
F
